# INT-1B3, an LNP formulated miR-193a-3p mimic, promotes anti-tumor immunity by enhancing T cell mediated immune responses via modulation of the tumor microenvironment and induction of immunogenic cell death

**DOI:** 10.18632/oncotarget.28608

**Published:** 2024-07-12

**Authors:** Chantal L. Duurland, Thijs de Gunst, Harm C. den Boer, Marion T.J. van den Bosch, Bryony J. Telford, Rogier M. Vos, Xiaolei Xie, Mingfa Zang, Fang Wang, Yingying Shao, Xiaoyu An, Jingjing Wang, Jie Cai, Ludovic Bourré, Laurens A.H. van Pinxteren, Roel Q.J. Schaapveld, Michel Janicot, Sanaz Yahyanejad

**Affiliations:** ^1^InteRNA Technologies BV, Utrecht, The Netherlands; ^2^Crown Bioscience Inc., San Diego, CA 92127, USA

**Keywords:** miR-193a-3p, microRNA mimic, T cell mediated immunity, immunogenic cell death, cancer

## Abstract

microRNAs (miRNAs) are small, non-coding RNAs that regulate expression of multiple genes. MiR-193a-3p functions as a tumor suppressor in many cancer types, but its effect on inducing specific anti-tumor immune responses is unclear. Therefore, we examined the effect of our lipid nanoparticle (LNP) formulated, chemically modified, synthetic miR-193a-3p mimic (INT-1B3) on anti-tumor immunity. INT-1B3 inhibited distant tumor metastasis and significantly prolonged survival. INT-1B3-treated animals were fully protected against challenge with autologous tumor cells even in absence of treatment indicating long-term immunization. Protection against autologous tumor cell challenge was hampered upon T cell depletion and adoptive T cell transfer abrogated tumor growth. Transfection of tumor cells with our miR-193a-3p mimic (1B3) resulted in tumor cell death and apoptosis accompanied by increased expression of DAMPs. Co-culture of 1B3-transfected tumor cells and immature DC led to DC maturation and these mature DC were able to stimulate production of type 1 cytokines by CD4+ and CD8+ T cells. CD4-CD8- T cells also produced type 1 cytokines, even in response to 1B3-transfected tumor cells directly. Live cell imaging demonstrated PBMC-mediated cytotoxicity against 1B3-transfected tumor cells. These data demonstrate for the first time that miR-193a-3p induces long-term immunity against tumor development via modulation of the tumor microenvironment and induction of immunogenic cell death.

## INTRODUCTION

The treatment landscape for cancer has changed a lot over the past few years. Despite exciting advancement in immune checkpoint therapies and development of novel small molecules that target critical cancer survival pathways, only a fraction of patients responds to these therapies. One of the main limitations of current therapeutics is their single target approach [1, 2]. In this regard, microRNAs (miRNAs) represent a promising new therapeutic approach. MiRNAs are endogenous, naturally occurring, small non-coding RNAs, approximately 19–25 nucleotides in length, and bind their target messenger RNA (mRNA) via the 3′ untranslated regions (UTRs) leading to mRNA degradation or translational repression. Each individual miRNA targets many different mRNAs [3], and thus multiple genes and consequently multiple signaling pathways. Furthermore, miRNAs play a critical role in pathogenesis of many diseases including cancer by regulating multiple cancer hallmarks, e.g., immune surveillance [4–7].

MiR-193a-3p functions as a tumor suppressor in several cancer types, e.g., melanoma, colorectal, breast, lung, liver, and hematological cancers [8, 9]. Many studies including our own findings reported a significant role for miR-193a-3p in inhibiting tumor growth via targeting critical genes involved in cell cycle arrest, apoptosis, senescence and DNA damage, but also migration, invasion and metastasis [9]. Additionally, predicted target genes of miR-193a-3p identified by programs such as Target Scan and our own transcriptome analysis [10] include genes affecting immune cell behavior, e.g., priming, activation and proliferation of T cells. This led to the hypothesis that in addition to its effect on tumor cells, miR-193a-3p also affects immune cells and potentially induces an anti-tumor immune response.

In this study, we examined the effect of our lipid nanoparticle (LNP) formulated, chemically modified, synthetic miR-193a-3p mimic (INT-1B3) on animal survival, tumor microenvironment (TME) and induction of anti-tumor immune responses. Our data show that systemic administration of INT-1B3 to 4T1 and H22 tumor bearing immunocompetent mice prolonged animal survival by reducing metastasis compared to phosphate buffered saline (PBS) or anti-programmed death (PD) 1 treatment. Long-term animal survival was T cell dependent and tumors from INT-1B3-treated mice showed an increase in effector CD8+ T cells, and reduction in regulatory T cells (Treg) and monocytic myeloid-derived suppressor cells (mMDSC). In addition, 1B3 induces immunogenic cell death as demonstrated by increased expression of damage-associated molecular patters (DAMPs) that in turn lead to maturation of dendritic cells (DC) which can activate type 1 cytokine producing CD4+, CD8+ and CD4-CD8- T cells leading to peripheral blood mononuclear cell (PBMC)-mediated cytotoxicity.

## RESULTS

### INT-1B3 requires adaptive immunity to suppress tumor regrowth and spontaneous metastasis leading to prolonged animal survival

**Figure 1:**
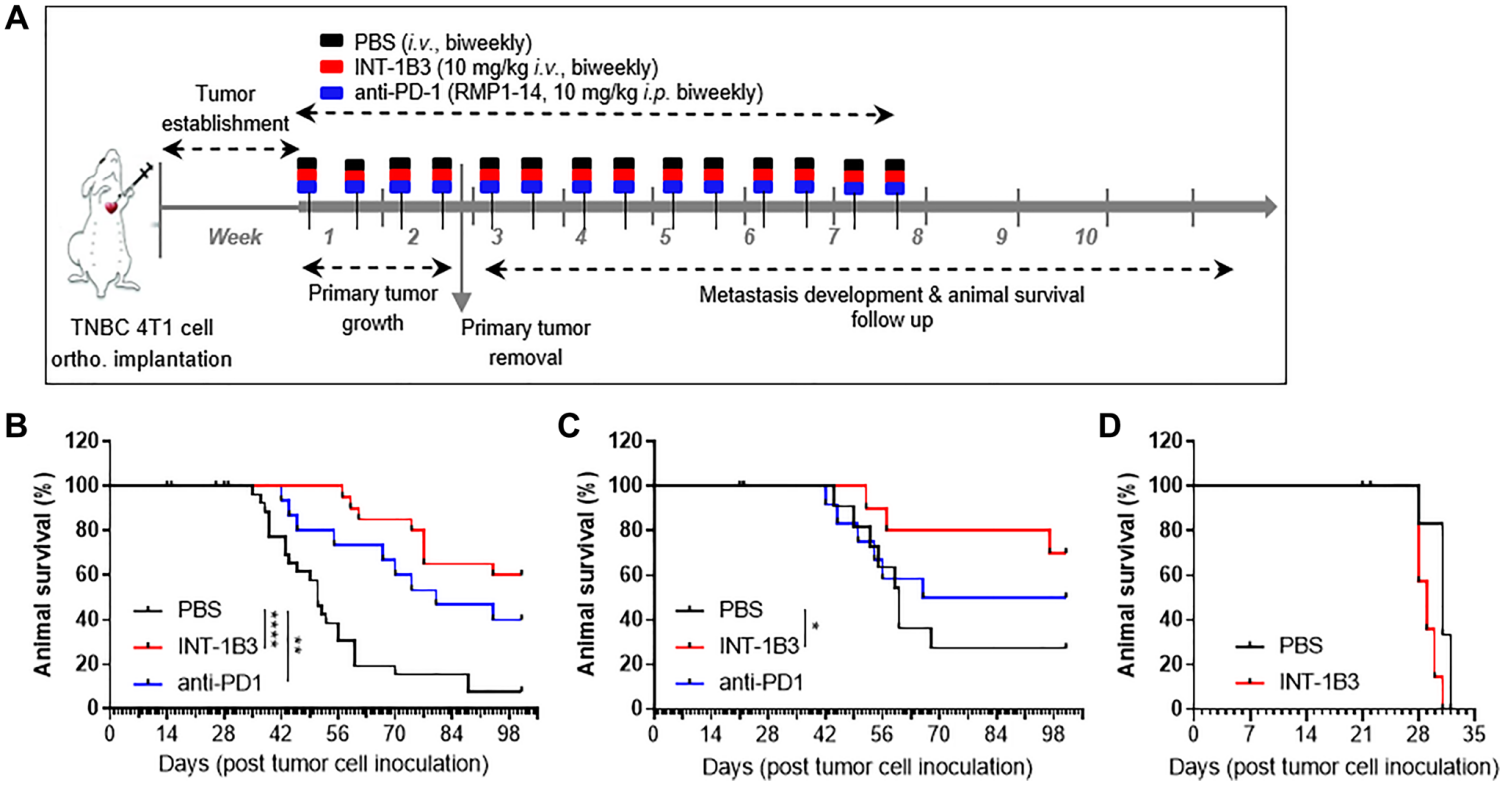
INT-1B3 requires adaptive immunity to induce a long-term anti-tumor response against 4T1 tumors. (**A**) Schematic representation of the experimental setup used. Balb/c mice were inoculated orthotopically in the mammary fat pad with murine 4T1 cells (3 × 10^5^). Treatment was initiated when tumors reached a volume of ~100 mm^3^ (established tumor). PBS, INT-1B3 (10 mg/kg, i.v.) and anti-PD1 (10 mg/kg, i.p) were administered biweekly for up to seven weeks. Primary tumors were surgically removed when the average tumor volume in each treatment group reached ~800 mm^3^. (**B**, **C**) Kaplan-Meier plots showing percentage survival per group at indicated time points for study #1 (B) (*n* = 12 per group) and study #2 (C) (*n* = 30 for PBS and INT-1B3, *n* = 15 for anti-PD1). (**D**) Murine 4T1 cells (3 × 10^5^) were inoculated orthotopically in the mammary fat pad of immunodeficient SCID Beige mice. Mice were treated as explained above with PBS and INT-1B3 (*n* = 8 for PBS, *n* = 16 for INT-1B3). Kaplan-Meier plot showing percentage survival per group at indicated time points. Significance was analyzed compared to PBS, ^*^ *p* < 0.05, ^**^ *p* < 0.01, ^****^ *p* < 0.0001.

To investigate the anti-tumor effect of INT-1B3, immunocompetent mice with established 4T1 tumors in the mammary fat pad were randomized into two study cohorts and three study arms per cohort, each of which was treated with INT-1B3, anti-PD1 and PBS on a biweekly schedule for up to seven weeks (Figure 1A).

In the first cohort, primary tumor growth was studied and INT-1B3 treatment did not reveal significant tumor growth delay compared to PBS (Supplementary Figure 1A). To allow development of metastasis, primary tumors from mice in the second cohort were surgically removed when the average tumor volume reached ~800 mm^3^ in each treatment group. Remarkably, INT-1B3 inhibited distant tumor metastasis in the lungs compared to anti-PD1 and PBS-treated mice (Supplementary Figure 1B, 1C) and significantly prolonged animal survival in two independent studies (Figure 1B, 1C). Interestingly, no effect of INT-1B3 was observed on animal survival of immune-deficient mice after inoculation with 4T1 tumor cells (Figure 1D) and all animals developed distant lung metastases (not shown). This data suggests that animal survival is strongly linked to the host immune system and that INT-1B3 can modulate the immune system leading to reduced tumor regrowth and metastasis, and thereby prolongs animal survival to a greater extent than anti-PD1.

Uptake of INT-1B3 by tumor and liver cells following INT-1B3 administration to 4T1 tumor-bearing Balb/c mice was measured by quantifying miR-193a-3p/1B3 levels. Interestingly, 1B3 levels in the tumor remained stable for up to 72 hours after the last INT-1B3 administration (Supplementary Figure 2A, 2B). mRNA expression of target genes *Entpd1* in tumor and *Dcaf7* in liver was reduced indicating biological activity of INT-1B3 (Supplementary Figure 2C, 2D). These genes were previously identified as targets of miR-193a-3p by Target Scan and our own transcriptome analysis [10].

### The anti-tumor response induced by INT-1B3 is T cell dependent

**Figure 2:**
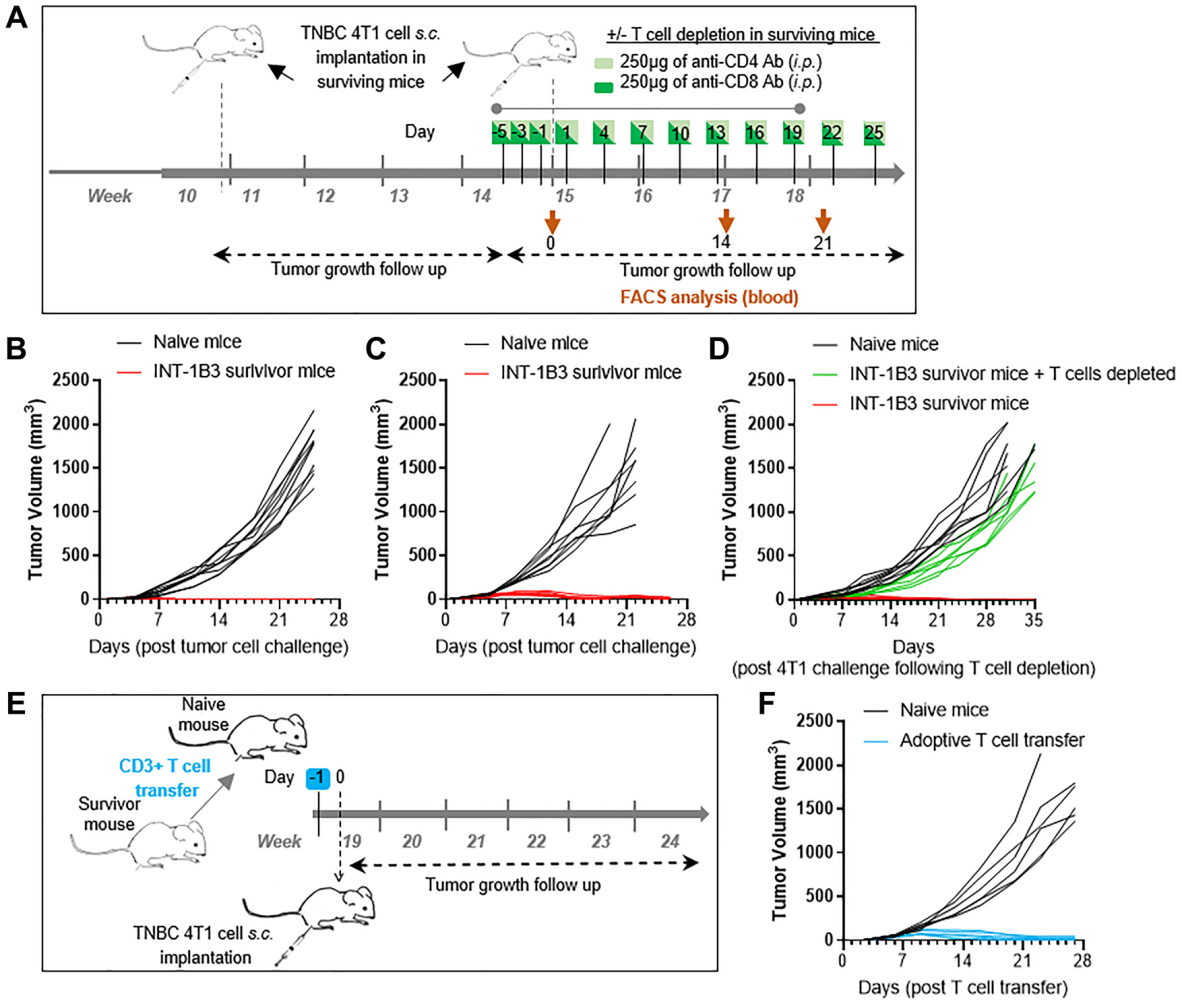
The anti-tumor response induced by INT-1B3 is T cell dependent. (**A**) Schematic representation of the continued experimental setup from Figure 1A. Naive, age-matched BALB/c mice and surviving INT-1B3-treated animals were ‘challenged’ by subcutaneous (s.c.) injection of autologous 4T1 tumor cells (3 × 10^5^) in the flank. (**B**, **C**) Graphs showing tumor growth over time in the absence of any further treatment for study #1 (B) (*n* = 7–8 per group) and study #2 (C) (*n* = 10 for naive mice, *n* = 19 for INT-1B3 survivor mice, 5/19 INT-1B3 survivor mice died on various days post tumor cell challenge, but not due to a palpable tumor). (**D**) T cells were depleted in INT-1B3-survivor mice by repeated administration of anti-CD4 and anti-CD8 antibodies (i.p., 250 μg), see Figure 2A. After one week of antibody administration on a every other day schedule, INT-1B3-survivor mice treated with and without anti-CD4 and anti-CD8 antibodies and naive, age-matched mice were re-challenged (s.c.) with autologous 4T1 tumor cells (3 × 10^5^) in the flank (*n* = 7–8 per group). Graph showing tumor growth over time after 4T1 challenge following T cell depletion. (**E**) Schematic representation of the continued experimental setup from Figure 2A. CD3^+^ T cells were isolated from spleen and lymph nodes of surviving INT-1B3-treated 4T1-challenged mice. Pooled CD3+ T cells (1 × 10^7^) were injected i.v. into age-matched, naive mice. One day after T cell transfer, naive mice were s.c. inoculated with 4T1 tumor cells (3 × 10^5^). (**F**) Graph showing tumor growth over time after T cell transfer (*n* = 6 per group).

To investigate whether INT-1B3-treated mice acquired protective immunity against 4T1 tumor cells, survivor mice previously treated with INT-1B3 (INT-1B3 survivor mice), or age-matched, naive mice were challenged by subcutaneous injection of 4T1 cells 10 weeks after start of treatment (~3 weeks after final INT-1B3 administration). Tumor growth was monitored in absence of any further treatment (Figure 2A). Interestingly, while age-matched, naive mice showed rapid 4T1 tumor growth within four weeks in two independent studies, INT-1B3 survivor mice showed initial tumor growth up to ~70 mm^3^ within the first week followed by complete tumor regression (Figure 2B, 2C) indicating INT-1B3-induced long-term immunization.

To determine whether the long-term immunity observed in INT-1B3-treated mice was T cell mediated, 4T1-challenged INT-1B3 survivor mice were treated with anti-CD4 and anti-CD8 antibodies before rechallenge with 4T1 tumor cells (Figure 2A). Depletion of CD4+ and CD8+ cells was confirmed by flow cytometry (Supplementary Figure 3A–3C). Again, age-matched, naive mice showed rapid 4T1 tumor growth, while INT-1B3 survivor mice showed initial tumor growth up to ~80 mm^3^ within the first week followed by complete tumor regression. Strikingly, protection against 4T1 rechallenge was markedly hampered upon depletion of CD4+ and CD8+ cells (Figure 2D). Furthermore, adoptive transfer of CD3+ T cells isolated from rechallenged, INT-1B3-survivor mice into age-matched, naive mice completely abrogated 4T1 tumor growth and led to full protection (Figure 2E, 2F). Thus, treatment with INT-1B3 leads to an acquired, long-term, and transferable T cell mediated immunity in 4T1 tumor-bearing mice.

### INT-1B3 also induces long-term anti-tumor responses in mice bearing syngeneic H22 liver tumors

**Figure 3:**
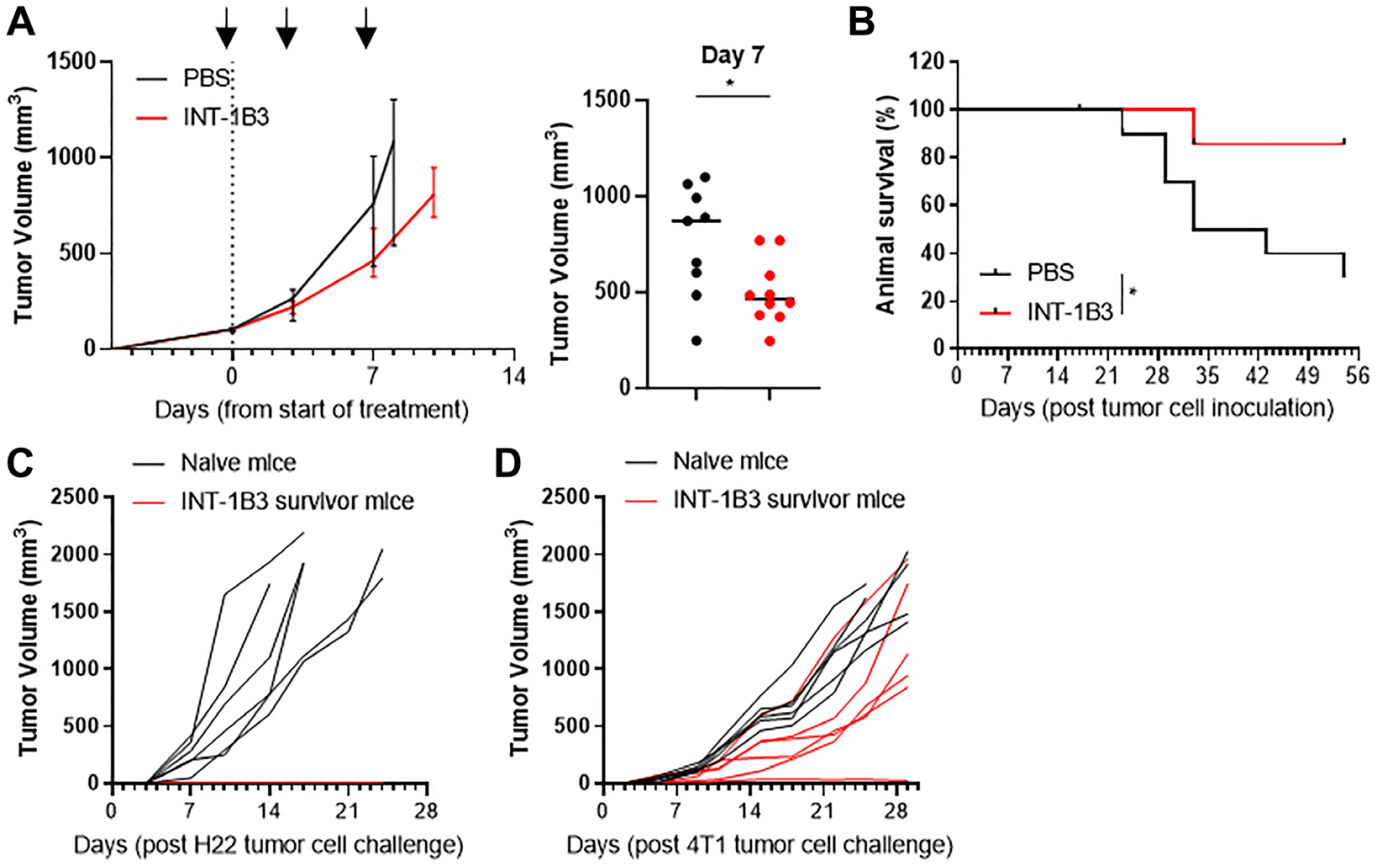
INT-1B3 also induces long-term anti-tumor responses in mice bearing syngeneic H22 liver tumors. Balb/c mice were inoculated s.c. with murine H22 cells (1 × 10^6^). Treatment was initiated when tumors reached a volume of ~100 mm^3^ (established tumor, dashed line). PBS and INT-1B3 (10 mg/kg, i.v.) were administered biweekly (black arrows) for up to two weeks. (**A**) Graphs showing primary tumor volume (mm^3^) over time up until primary tumor removal and individual tumor volumes (mm^3^) for each treatment group on day 7 (*n* = 10 per group). Lines indicate median with interquartile range. (**B**) Primary tumors were surgically removed when average tumor volumes in each group reached ~800 mm^3^, and animal survival was followed in absence of further treatment. Three mice in the INT-1B3 group died due to surgery and were excluded. Kaplan-Meier plot showing percentage survival per group at indicated time points (*n* = 10 per group). (**C**) Naive, age-matched mice and surviving INT-1B3-treated mice were ‘challenged’ by s.c. injection of autologous H22 tumor cells (1 × 10^6^) in the flank. Graph showing tumor growth over time in the absence of any treatment (*n* = 6 per group). (**D**) Naive, age-matched mice and surviving INT-1B3-treated mice were ‘challenged’ by s.c. injection of heterologous 4T1 tumor cells (3 × 10^5^) in the opposite flank of the animals. Graph showing tumor growth over time (*n* = 6 per group). Significance was analyzed compared to PBS, ^*^ *p* < 0.05.

To extend our findings, the effect of INT-1B3 on anti-tumor response was also investigated in syngeneic mice bearing H22 liver tumors. The study design was similar to the 4T1 model, but mice were only treated biweekly with INT-1B3 and PBS until surgical resection of primary tumors. The impact of changing the schedule of administration on animal survival was minimal. INT-1B3 treatment up to surgical removal of primary tumors, or up to two weeks or four weeks after surgery resulted in a comparable improvement of animal survival (Supplementary Figure 4). In the H22 model, INT-1B3 treatment revealed a strong albeit not significant primary tumor growth delay compared to PBS-treated mice over time (Figure 3A). After removal of primary tumors, INT-1B3-treated mice showed significantly prolonged survival (Figure 3B) indicating that the INT-1B3-induced anti-tumor response is not restricted to 4T1 tumors. To examine whether the anti-tumor response was maintained long-term, INT-1B3 survivor mice were challenged ~6 weeks post-final INT-1B3 administration with H22 tumor cells. While naive mice showed rapid H22 tumor growth, INT-1B3-treated mice did not form a palpable tumor (Figure 3C). This protection was impaired upon rechallenge of surviving H22-challenged mice with heterologous 4T1 tumor cells (Figure 3D).

### INT-1B3 enhances anti-tumor immunity by modulating immune cell composition of the tumor microenvironment

**Figure 4:**
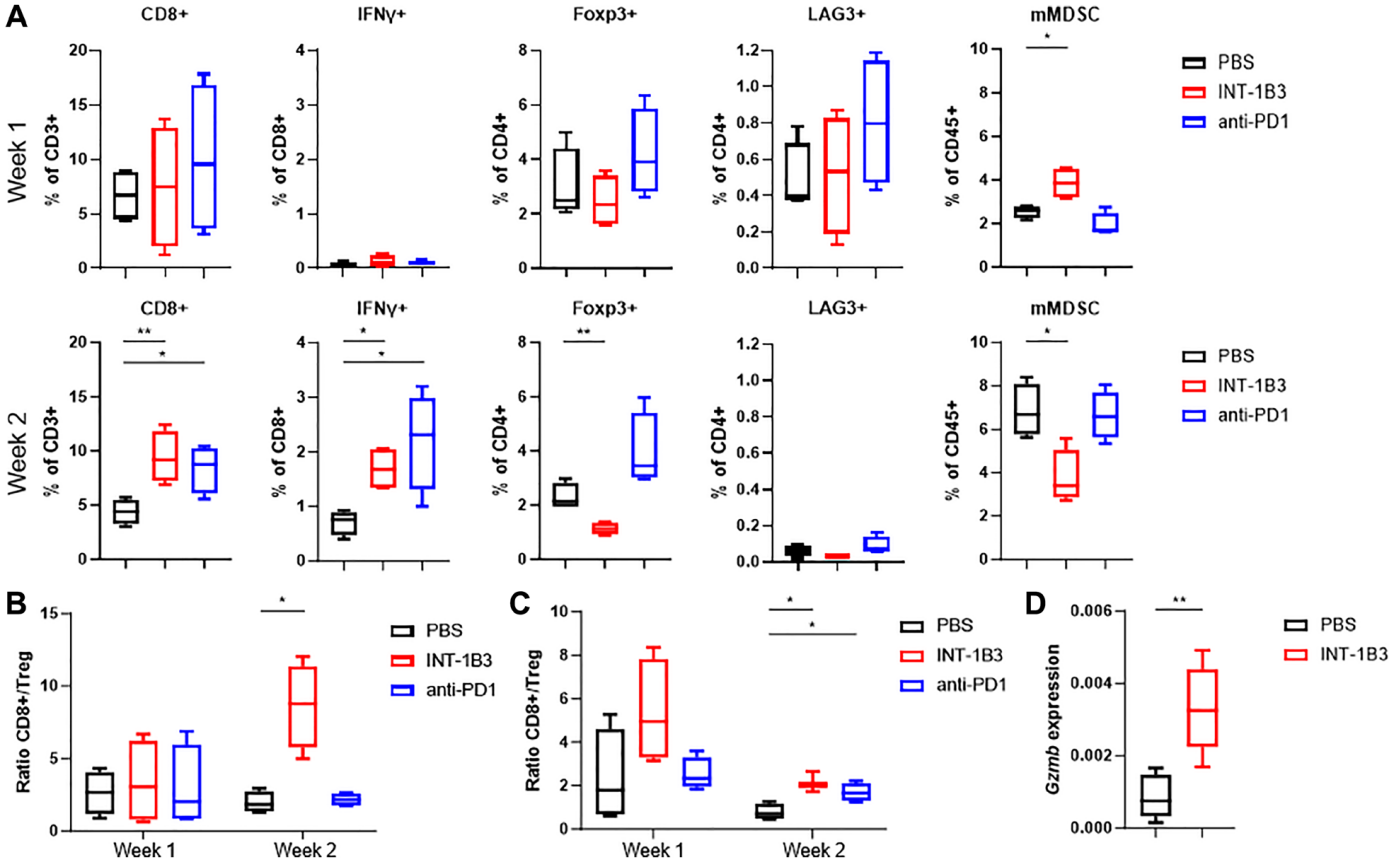
INT-1B3 enhances anti-tumor immunity by modulating immune cell composition of the tumor microenvironment. Balb/c mice were inoculated orthotopically in the mammary fat pad with murine 4T1 tumor cells (3 × 10^5^). Treatment was initiated when tumors reached a volume of ~100 mm^3^ (established tumor). PBS, INT-1B3 (10 mg/kg, i.v.), and anti-PD1 (10 mg/kg, i.p.) were administered biweekly for up to two weeks (*n* = 4 per group). Mice were euthanized two days after the last injection upon one (week 1) or two (week 2) weeks of treatment, and tumors were harvested for analysis. (**A**) Summary graphs showing percentage CD8+ of CD3+, IFNγ+ of CD8+, Foxp3+ of CD4+, LAG3+ of CD4+, and mMDSC in tumors from indicated groups. T cell populations were gated on CD45+CD3+ cells before further analysis of CD4+ and CD8+ populations, and mMDSC were defined as percentage CD11b+Gr-1^hi+dim^ of CD45+. (**B**, **C**) Summary graphs showing ratio of CD8+ to Treg in 4T1 (B) and H22 (C) tumors after one or two weeks of treatment. (**D**) mRNA expression of *Gzmb* in 4T1 tumors collected in after two weeks of indicated treatment (*n* = 6 per group). Significance was analyzed compared to PBS, ^*^ *p* < 0.05, ^**^ *p* < 0.01. In all graphs, the whiskers indicate min-max and the lines indicate the median.

Next, the effect of INT-1B3 on immune cell composition of the TME was investigated in parallel with anti-PD1. Analysis of T cell subsets after two weeks of treatment showed a significant increase in percentage CD8+ T cells while percentage Foxp3+ of CD4+ T cells, Treg, was significantly decreased. Interestingly, the percentage interferon gamma (IFNγ) producing CD8+ T cells was increased after two weeks of treatment. Despite an increased percentage CD8+ T cells and enhanced IFNγ production by CD8+ T cells in anti-PD1-treated mice, the level of Treg was also significantly increased after two weeks (Figure 4A). Furthermore, the total MDSC population was increased upon INT-1B3 treatment (Supplementary Figure 5A), but its immunosuppressive monocytic subpopulation (mMDSC) was significantly decreased after two weeks. The levels of mMDSC remained unchanged upon anti-PD1 treatment. The percentage LAG3+ of CD4+ cells, LAG3 is a co-inhibitory receptor, was low in all groups (Figure 4A). Consistent with reduced tumor regrowth and increased survival, a time-dependent increase (4.7×) in the CD8+/Treg ratio, known to correlate with a favorable outcome [11], was observed upon INT-1B3 treatment compared to PBS and anti-PD1 (Figure 4B) suggesting that INT-1B3 therapy alone induces potent anti-tumor immunity in 4T1 tumors. Importantly, the effect of INT-1B3 on the CD8+/Treg ratio was confirmed in the H22 model (Figure 4C). The increase in percentage CD8+ T cells and IFNγ production were milder upon INT-1B3 treatment (partly attributed to bigger variations in control group), but levels of Treg and mMDSC were substantially decreased after two weeks (Supplementary Figure 6).

In addition, INT-1B3-treated tumors revealed a strong trend towards increased expression of *Cxcl5*, *Cxlc9* and *Cxcl10* (Supplementary Figure 5B), T cell recruiting chemokines, which is consistent with increased levels of CD8+ T cells. Furthermore, granzyme B (*Gzmb*) expression was significantly increased in tumors from INT-1B3-treated mice (Figure 4D).

Moreover, the effect of INT-1B3 on expression of several predicted targets of miR-193a-3p was assessed. Expression of *Ezh2*, important in Treg stability and function [12–14], and *Entpd1* (gene encoding CD39), and percentage CD73+ of CD3+ cells, both critical enzymes involved in generation of immunosuppressive adenosine [15], were strongly downregulated confirming that a biologically/molecularly active drug concentration was achieved within the tumor and TME (Supplementary Figure 5C, 5D). Importantly, direct binding of miR-193a-3p/1B3 to the 3′UTR sequence of mouse Nt5e (gene encoding CD73) was demonstrated here for the first time validating Nt5e as a direct target of miR-193a-3p (Supplementary Figure 7A).

Taken together, these data provide evidence that treatment with INT-1B3 modulates the TME by recruiting CD8+ T cells that produce IFNγ and reducing infiltration of Treg and mMDSC.

### 1B3 induces upregulation of calreticulin and downregulation of CD73 on tumor cells

**Figure 5:**
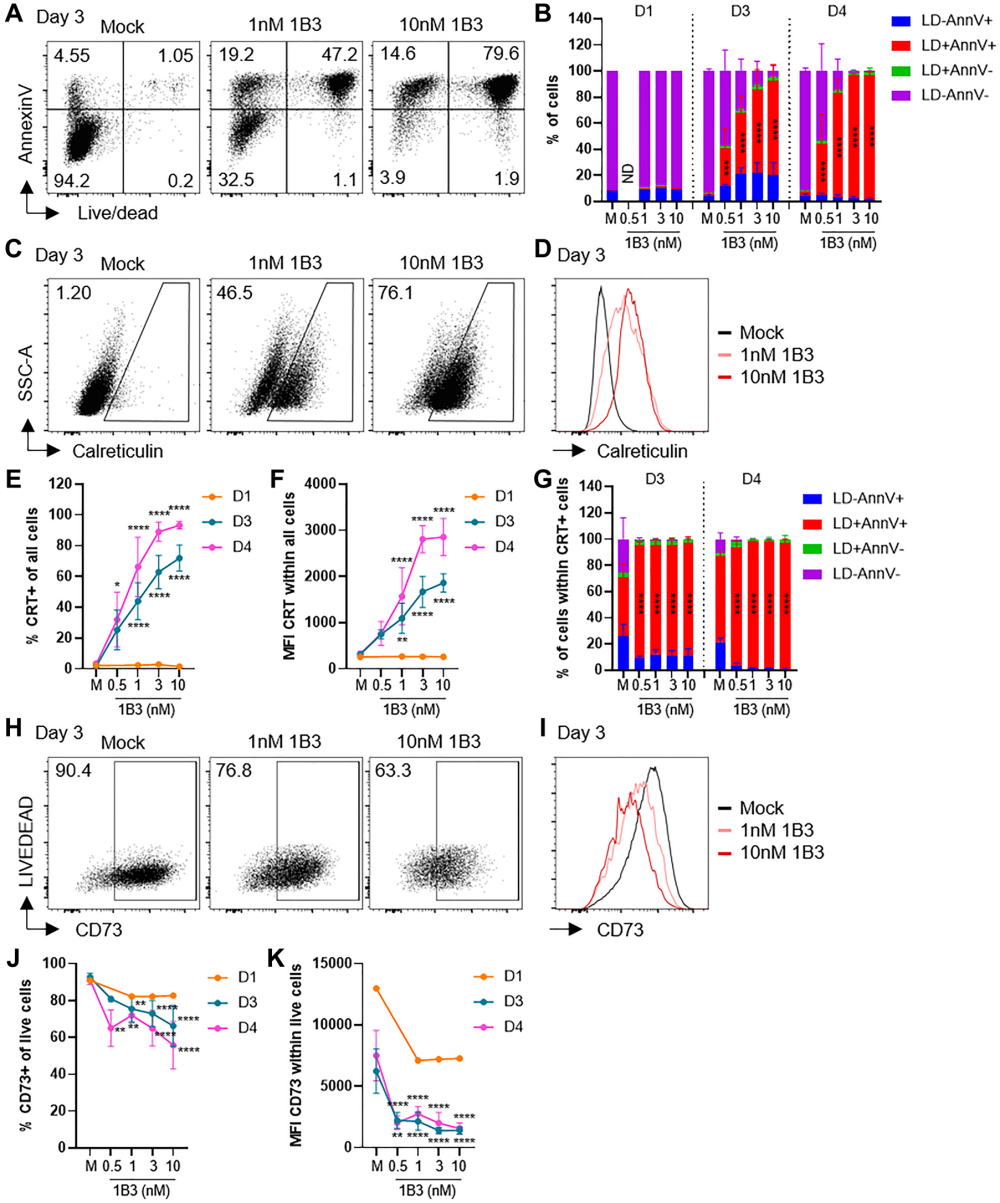
1B3 induces upregulation of calreticulin and downregulation of CD73 on tumor cells. HCT116 cells were transfected with 1B3 at indicated concentrations or mock (M) transfected and analyzed by flow cytometry at various timepoints (*n* = 1–6 independent experiments). (**A**) Representative dot plots showing live/dead (LD) against Annexin V (AnnV) on day 3. (**B**) Summary graph showing % LD-AnnV+ (early apoptotic cells), LD+AnnV+ (late apoptotic cells/dead cells), LD+AnnV- and LD-AnnV- (viable cells) cells. (**C**) Representative dots plots showing percentage calreticulin (CRT) positive cells on day 3. (**D**) Histogram overlay showing calreticulin expression on day 3. (**E**, **F**) Summary graphs showing percentage CRT+ cells (E) and MFI of CRT (F). (**G**) Summary graph showing % LD-AnnV+ (early apoptotic cells), LD+AnnV+ (late apoptotic cells/dead cells), LD+AnnV- and LD-AnnV- (viable cells) cells within CRT+ cells. (**H**) Representative dots plots showing percentage CD73+ cells gated on live cells on day 3. (**I**) Histogram overlay showing CD73 expression within live cells on day 3. (**J**, **K**) Summary graphs showing percentage CD73+ cells (J) and MFI of CD73 (K). All summary graphs show mean ± SD. ND = not determined. Significance analyzed compared to mock transfected cells per timepoint, ^*^ *p* < 0.05, ^**^ *p* < 0.01, ^***^ *p* < 0.001, ^****^ *p* < 0.0001.

To further define the potential mechanism by which INT-1B3 induces long-term immunity, 1B3, synthetic miR-193a-3p mimic, was used. Previously, we demonstrated that 1B3 had a strong effect on tumor cell proliferation and apoptosis [9] suggesting that INT-1B3 may induce an anti-tumor immune response via initiation of immunogenic cell death. Immunogenic cell death is a form of regulated cell death characterized by dying tumor cells expressing antigens and expression of DAMPs, e.g., calreticulin (CRT) and adenosine triphosphate (ATP), that induce antigen presentation and maturation of dendritic cells (DC) leading to anti-tumor immune responses [16–18]. The potential of 1B3 to induce immunogenic cell death was examined using the human colon cell line HCT116, because HCT116 was one the cell lines that previously showed the strongest reduction in cell proliferation and induction of apoptosis upon transfection with 1B3 [9]. Indeed, transfection of HCT116 with 1B3, but not 3A1 (irrelevant miRNA mimic), induced cell death and apoptosis in a dose-dependent manner compared to mock transfected cells (Figure 5A, 5B; Supplementary Figure 8A). In addition, 1B3 induced a dose- and time-dependent increase in percentage CRT+ cells and median fluorescence intensity (MFI) of CRT compared to mock (Figure 5C–5F) whereas the effect of 3A1 was comparable to mock (Supplementary Figure 8B, 8C). The increase in CRT levels was related to induction of cell death and apoptosis by 1B3, because the CRT+ population consisted mainly of early apoptotic and death/late apoptotic cells (Figure 5G). ATP is converted into adenosine by CD39 (ATP to ADP, ADP to AMP) and CD73 (AMP to adenosine) [15]. The effect of 1B3 on ATP levels was analyzed using CD73 protein expression, *NT5E* expression, adenosine levels and free phosphate levels as a surrogate for ATP levels. Consistent with direct binding of 1B3 to mouse *Nt5e*, we demonstrated for the first time that miR-193a-3p also binds directly to human *NT5E* (Supplementary Figure 7A). In addition, 1B3 reduced *NT5E* expression in several human cancer cell lines (Supplementary Figure 7B) and there was a strong reduction in percentage CD73+ cells and MFI CD73 compared to mock and 3A1-transfected cells (Figure 5H–5K and Supplementary Figure 8D, 8E). The effect of 1B3 on CD73 protein expression was also confirmed by western blot (Supplementary Figure 7C and Supplementary Table 1). Furthermore, adenosine and free phosphate levels were reduced upon 1B3 transfection, and this effect was comparable to the effect of si*NT5E* suggesting that ATP levels might be higher in 1B3-transfected conditions (Supplementary Figure 7D, 7E). These data indicate that 1B3 induces cell death, apoptosis and expression of DAMPs, and thus potentially immunogenic cell death.

### 1B3-transfected tumor cells induce DC maturation and these DC can activate CD4+ and CD8+ T cells, but also CD4-CD8- T cells

**Figure 6:**
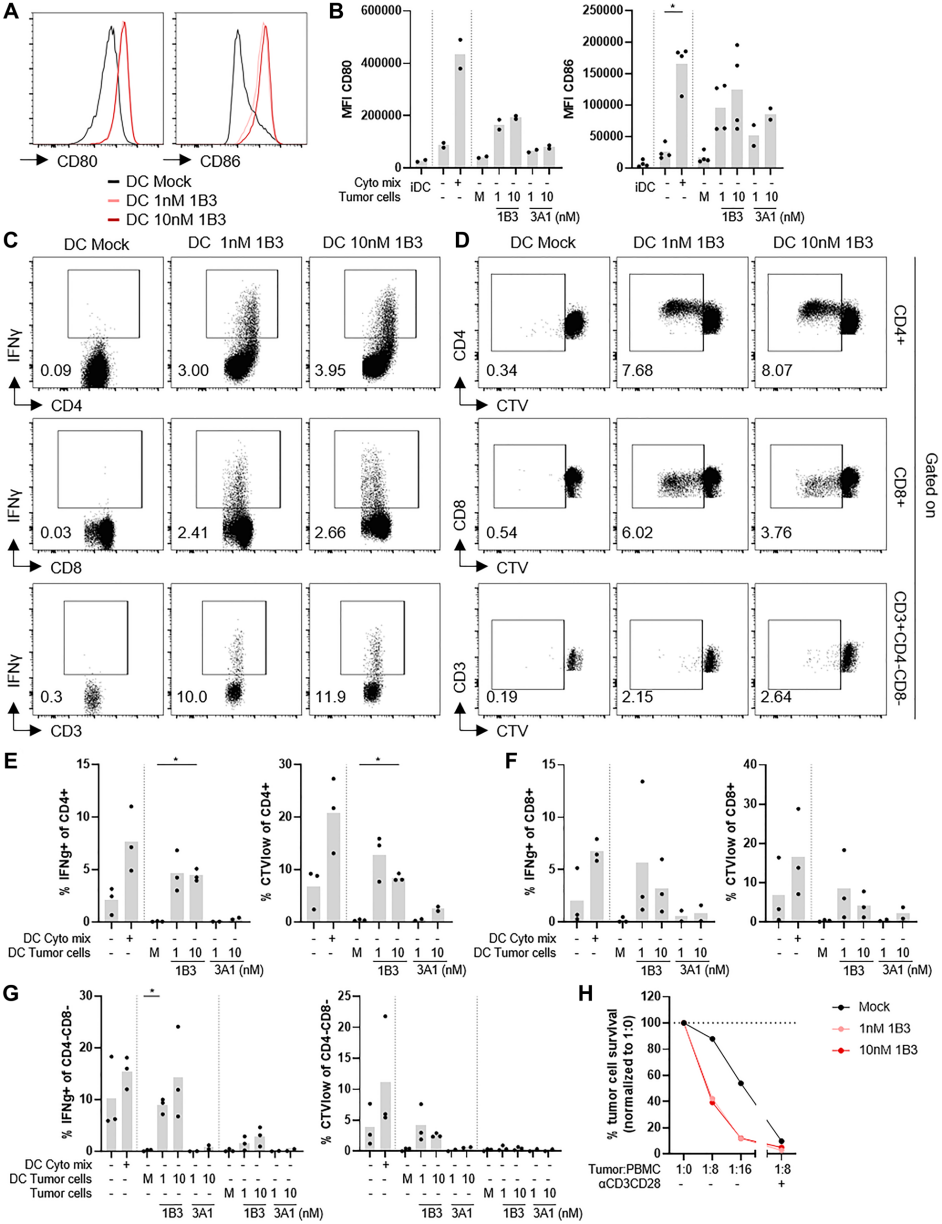
1B3 transfected tumor cells induce DC maturation and these DC can activate CD4+ and CD8+ T cells, and CD4-CD8- T cells. (**A**, **B**) HCT116 cells were transfected with 1B3 or 3A1 at indicated concentrations or cells were mock (M) transfected and cultured for 72 hours. CD14+ cells were isolated from PBMC of healthy donors and cultured in presence of rhIL-4 and rhGM-CSF to induce differentiation into immature DC (iDC) for 6 days. After 6 days, immature DC were harvested and added to transfected tumor cells for 24 hours to induce DC maturation. Immature DC were also cultured in presence or absence of a cytokine cocktail (cyto mix) known to induce DC maturation as a control. Cells were analyzed for expression of DC maturation markers by flow cytometry before and after maturation (*n* = 2–4). Histogram overlays and summary graphs showing MFI of CD80 and CD86 within CD45+ cells. (**C**–**F**) CTV-labelled PBMC were co-cultured for 5 days with DC matured by co-culture with HCT116 transfected with mock, 1B3 or 3A1 at a ratio of DC:PBMC of 1:10 (*n* = 2–3). (C, D) Representative dot plots showing percentage IFNγ+ (C) or CTVlow (D) of CD4+ cells (top), CD8+ (middle), and CD4-CD8- (bottom) T cells. (E, F) Summary graphs showing percentage IFNγ+ and CTVlow of CD4+ cells (E) and CD8+ T cells (F). (**G**) CTV-labelled PBMC were co-cultured for 5 days with DC matured by co-culture with HCT116 transfected with mock, 1B3 or 3A1 at a ratio of DC:PBMC of 1:10, or with transfected HCT116 cells. Summary graphs showing percentage IFNγ+ and CTVlow of CD4-CD8- T cells. (**H**) NucLight Red labelled HCT116 cells were transfected overnight with 1B3 at indicated concentrations or mock transfected. After overnight transfection, cells were harvested, counted, reseeded into 96-well plates and allowed to adhere to the plate for 20–24 hours before adding PBMC. PBMC were added at ratio tumor cells:PBMC of 1:0, 1:8, 1:16 or 1:8 + aCD3CD28. Summary graph shows percentage tumor cell survival when normalized to tumor:PBMC of 1:0 (no PBMC) condition from one representative of 3 independent experiments. ^*^ *p* < 0.05. Bars in summary graphs represent mean.

To determine whether the effect of 1B3 on expression of DAMPs affects DC maturation, monocyte-derived DC (moDC) were added to HCT116 cells transfected with mock, 1B3 or 3A1. moDC were CD209+ and HLA-DR, DP, DQ+ before co-culture with transfected tumor cells and levels were not affected upon co-culture (Supplementary Figure 9A, 9B). Furthermore, MFI of CD80 and CD86, and percentage CD86+ cells were low on these cells before co-culture with transfected tumor cells confirming that at this stage cells were immature DC (iDC). MFI of CD80 and CD86 were increased upon co-culture with 1B3-transfected tumor cells compared to co-culture with mock transfected tumor cells, but the effect was not as strong as when cells were stimulated with a cytokine cocktail known to induce DC maturation [19, 20]. A similar effect was observed for percentages CD80+ and CD86+ cells and these levels were comparable to the levels induced by the cytokine cocktail. Co-culture with 3A1-transfected tumor cells also showed some increase in expression of CD80 and CD86 (Figure 6A, 6B and Supplementary Figure 9C, 9D).

Next, the T cell stimulatory capacity of DC matured by co-culture with mock, 1B3 or 3A1-transfected HCT116 was examined by adding CellTrace Violet (CTV)-labelled PBMC to co-cultures of DC and transfected tumor cells. DC matured by co-culture with 1B3-transfected HCT116 induced production of the type 1 cytokines IFNγ and TNFα, and proliferation (expressed as percentage CTVlow cells) by CD4+, CD8+, and CD4-CD8- T cells whereas DC matured by mock or 3A1-transfected tumor cells had no or limited effect (Figure 6C–6G; Supplementary Figure 9E). Stimulation of PBMC with 1B3-transfected tumor cells alone did not result in activation of CD4+ or CD8+ T cells, but CD4-CD8- T cells did show an increase in percentage IFNγ+ and TNFα+ cells suggesting that this population can respond to tumor cells directly (Figure 6G, Supplementary Figure 9E–9G). In control conditions, PBMC stimulated with DC matured by cytokine cocktail or αCD3CD28-coated beads showed activation of CD4+, CD8+ and CD4-CD8- T cells (Supplementary Figure 9E–9H).

To quantify and visualize PBMC-mediated cytotoxicity against 1B3-transfected HCT116, PBMC and mock or 1B3-transfected HCT116 were co-cultured at different ratio’s and monitored using live-cell imaging. Addition of αCD3CD28 antibodies to co-cultures resulted in a strong reduction in percentage tumor cell survival indicating that PBMC activation is important for PBMC-mediated cytotoxicity. 1B3 transfection alone already had a strong effect on percentage tumor cell survival, but this effect was greatly enhanced in presence of PBMC (Figure 6H and Supplementary Figure 10).

Thus, 1B3-transfected tumor cells induce DC maturation which can activate not only CD4+ and CD8+ T cell responses, but also CD4-CD8- T cells, and thereby mediate PBMC-mediated cytotoxicity against 1B3-transfected HCT116.

## DISCUSSION

**Figure 7:**
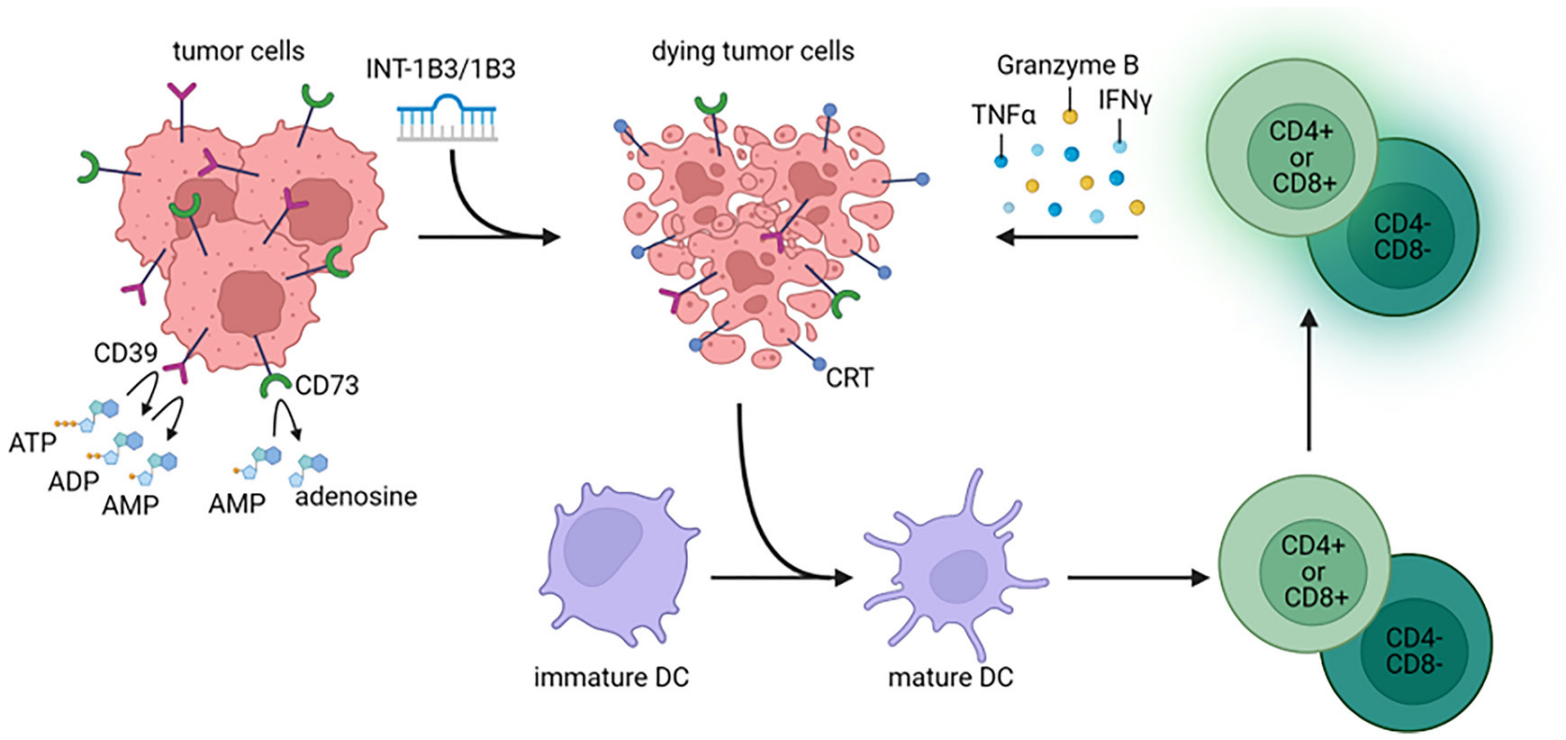
Proposed mode of action of INT-1B3/1B3. INT-1B3/1B3 induces apoptosis and cell death leading to expression of DAMPs, e.g., expression of calreticulin, reduced expression of CD39 and CD73 and thus reduced levels of ATP. Release of DAMPs induces maturation of DC and these DC can activate CD4+, CD8+ and CD4-CD8- T cells upon stimulation. Activated CD4+, CD8+ and CD4-CD8- T cells produce IFNγ, TNFα and granzyme B which leads to cytotoxicity against tumor cells. This illustration was created with https://www.biorender.com/.

The principle that miRNAs modulate multiple molecular pathways concurrently and thereby regulate important cellular processes highlights them as a potential novel therapeutic modality against cancer. In this study, we demonstrated that systemic administration of INT-1B3 in immunocompetent mice bearing 4T1 or H22 tumors reduced development of metastasis and prolonged animal survival. Surviving animals were fully protected against autologous tumor cell challenge and this protection was T cell dependent. Consistently, a time-dependent influx of effector CD8+ T cells and reduction in immunosuppressive cells within the TME was demonstrated. Furthermore, 1B3 triggers immunogenic cell death via induction of apoptosis and upregulation of DAMPs. 1B3-transfected tumor cells were able to induce DC maturation and thereby activated CD4+ and CD8+ T cells, but also CD4-CD8- T cells, leading to PBMC-mediated cytotoxicity against 1B3-transfected tumor cells (Figure 7).

The data presented here supports our previous findings demonstrating anti-tumor activity by INT-1B3 as single agent in experimental human Hep3B and A2058 tumor models [9]. Others reported that systemic administration of miR-193a-3p utilizing epoxide-derived lipidoid C12-200 nanoparticles into mice bearing orthotopic patient-derived triple-negative breast cancer xenografts strongly inhibited tumor progression [4]. Interestingly, while INT-1B3 reduced tumor regrowth and metastasis, and prolonged survival in immunocompetent mice, it had no effect in immunodeficient mice. This suggests that INT-1B3 requires a functional immune system to improve animal survival. Indeed, INT-1B3-treated mice showed full tumor regrowth upon tumor rechallenge when T cells were depleted and adoptive T cell transfer from immunized mice into naive mice completely abrogated autologous tumor growth leading to full protection highlighting the importance of T cells in the development of a long-term anti-tumor response. Several miRNAs have been linked to activation of T cell dependent anti-tumor immunity [21–24], but our study is the first to unveil such a role for miR-193a-3p.

Our previous studies demonstrated that 1B3 suppresses tumor cell growth and migration via multiple mechanisms including induction of cell death and apoptosis [9, 10]. Regulated cell death has long been considered an immunologically silent event, but over the years it has become clear that it can drive adaptive immunity depending on sufficient antigenicity and adjuvanticity [16–18]. Here, we confirmed that 1B3 induces apoptosis of tumor cells, but also induces expression of DAMPs required for DC maturation. DC matured in presence of dying tumor cells and cell surface expressed or secreted DAMPs can stimulate type 1 cytokine production and proliferation by CD4+ and CD8+ T cells. Interestingly, CD4-CD8- T cells also produced type 1 cytokines and even showed direct responses against 1B3-transfected tumor cells. Typically, CD4-CD8- T cells represent either natural killer (NK) T or γδ T cells, but further research is required to confirm this. γδ T cells can directly kill tumor cells in a major histocompatibility complex (MHC)-independent manner [25] while NK T cells can be activated in a CD1d-dependent or -independent manner [26] and this could explain the direct response of CD4-CD8- T cells against 1B3-transfected tumor cells. Co-culture of 1B3-transfected tumor cells and human PBMC demonstrated an additional effect of PBMC on top of the effect of 1B3 on tumor cell survival supporting immunogenic cell death as a potential mechanism for the anti-tumor response upon INT-1B3 treatment. Various miRNAs were reported to affect expression of DAMPs [27], but this is the first report showing that miR-193a-3p induces immunogenic cell death as a consequence of tumor cell death.

MiRNAs affect expression of multiple mRNAs and therefore we cannot exclude the possibility that INT-1B3 induces anti-tumor responses by targeting other mechanisms. Target prediction software programs and our own transcriptome analysis [10] identified a pool of immune related genes as miR-193a-3p targets including *NT5E*, *EZH2*, *PI3K*, *CDK4/6*. CD73 converts AMP to adenosine [15] and plays an important role in regulating tumor immunosurveillance and metastasis [28]. Adenosine is immunosuppressive and profoundly inhibits anti-tumor T cells, and is therefore a promising therapeutic target [29, 30]. We are the first to show that the gene encoding both mouse and human CD73 is a direct target of miRNA-193a-3p/1B3 and thus INT-1B3/1B3 reduces adenosine generation by directly affecting the functionality of CD73. In addition, expression of *Ezh2* was reduced in tumors from INT-1B3-treated mice. Pharmacological inhibition of EZH2 resulted in improved CD8+ T cell responses within tumors and was associated with reduced Foxp3 expression in Tregs [12–14]. Furthermore, *Ezh2*-deficient Tregs acquired a pro-inflammatory phenotype that supported stronger anti-tumor immunity [13]. Others demonstrated that abemaciclib (CDK4/CDK6 inhibitor) significantly enhanced anti-tumor responses in cell lines, animal models and breast cancer patients by increasing the capacity of tumor cells to present antigen and reducing Treg levels resulting in decreased Treg/CD8 T-cell ratio [31, 32]. PI3K inhibitors promote anti-tumor responses in a T cell dependent manner [33] and eradicated 4T1 tumors by reducing levels of MDSC when combined with immune checkpoint inhibitors [34]. In line with this, our recent study identified 1B3 as a strong activator of the PTEN pathway, which downregulates (PI3K)-AKT signaling by targeting critical genes [10], suggesting an additional mechanism for INT-1B3 to exert an effect on the TME.

In conclusion, the data presented here supports a strong potential for INT-1B3 as monotherapy by triggering a long-term T cell mediated immune response against tumor antigens via the induction of immunogenic cell death and modulation of the TME. In addition, we hypothesize that INT-1B3 could be combined with immune checkpoint therapies to convert immunologically ‘‘cold’’ into ‘‘hot’’ tumors that do not or poorly respond to current treatments.

## MATERIALS AND METHODS

### Mice

Female BALB/c mice and SCID Beige were purchased from Shanghai Lingchang Bio-Technology Co. Ltd and animal experiments were performed at Crown Biosciences (Taicang, China). Mice (5 per cage) were housed under specific pathogen-free conditions at the animal facility. For animal experiments, 50% extra mice were included at the start of the study to account for 50% failure in tumor establishment. Reported sample size is the number of mice in each group after randomization. Study protocols and any amendments involving the care and use of animals for this study were reviewed and approved by the Institutional Animal Care and Use Committee (IACUC) of Crown Biosciences. All animal procedures were conducted in strict compliance with the regulations of the Association for Assessment and Accreditation of Laboratory Animal Care (AAALAC) for animal experimentation.

### Cell lines and human samples

The murine tumor cell lines 4T1 and H22 were cultured in RPMI (Lonza) supplemented with 10% fetal bovine serum (FBS, Sigma-Aldrich) and 1% Penicillin/Streptomycin (P/S, Thermo Fisher Scientific). The human tumor cell line HCT116 (colon, ATCC) was cultured as recommended by the supplier. For cell passaging, cells were harvested using TripLE Express (Thermo Fisher Scientific).

Human peripheral blood mononuclear cells (PBMC) were isolated from buffy coats (Sanquin) using SepMate tubes (Stemcell Technologies). Cells were cryopreserved and stored until use. PBMC were cultured in RPMI (Capricorn Scientific GmbH) supplemented with 10% FBS and 1% P/S (10% RPMI). All cells were cultured at 37°C and 5% CO_2_.

### Oligonucleotides

The miR-193a-3p mimic 1B3 and control oligonucleotide 3A1 (unrelated miRNA, based on Thermo Fisher #4464058) were manufactured by BioSpring GmbH. INT-1B3 was manufactured by Axolabs GmbH. The nucleotide sequences are reported in Supplementary Table 2 (35).

### Tumor cell inoculation, animal randomization and treatment

4T1 and H22 cells were harvested and counted before tumor inoculation. Balb/c or SCID Beige mice (6–8 weeks old) were inoculated orthotopically in the mammary fat pad with 3 × 10^5^ 4T1 tumor cells or upper right flank with 1 × 10^6^ H22 tumor cells in 0.1ml phosphate buffered saline (PBS). Animal randomization was performed using “Matched distribution” randomization using StudyDirectorTM software (version 3.1.399.19) when the mean tumor size reached ~100 mm^3^. After randomization, tumor volumes were measured twice per week in two dimensions using a caliper and expressed in mm^3^ using the formula: V = 0.5 a × b^2^ where a and b are the length and width of the tumor, respectively. Researchers performing animal experiments and study director were aware of group allocations. Treatments with PBS, INT-1B3 (10 mg/kg, i.v.) or anti-PD1 (10 mg/kg, i.p., BioXcell) started immediately after randomization on a biweekly schedule with for up to 7 weeks depending on mouse model, readout, and phase of the study. The detailed treatment schedule is explained in the result section.

To determine uptake of INT-1B3 by tumor and liver, immunocompetent Balb/c mice bearing 4T1 tumors were randomized and treated with PBS or 10 mg/kg/administration of INT-1B3 (i.v.) once per day for 2 days. Tumors and livers were collected at timepoints indicated after the 2nd administration (*n* = 4 mice per timepoint).

Animals were excluded from analysis if they died due to surgery (up to 5 days after surgery) or when humane endpoint was reached (see figure legends).

### T cell depletion

Anti-CD4+ (clone GK1.5) and anti-CD8+ (clone 2.43) antibodies (250 μg, BioXCell) were simultaneously administered (i.p.) in 200 μl PBS. Antibody treatment started one week before tumor cell inoculation on a every other day schedule in the first week followed by every three days for four weeks.

### Adoptive T cell transfer

Spleen, and auxiliary, brachial and inguinal lymph nodes were harvested from 1B3-survivor mice, processed into single cell suspensions and pooled. Briefly, spleen and lymph nodes were pushed through a 70 μm cell strainer, the strainer was flushed with PBS containing 2% FBS and cells were centrifuged. Next, cells were incubated with ACK lysing buffer (Thermo Fisher Scientific), washed with PBS containing 2% FBS and RPMI containing 2% FBS, filtered through a 40 μm cell strainer, centrifugated and counted. CD3+ T cells were isolated using magnetic beads according to the manufacturer’s protocol (Miltenyi Biotech) and 1 × 10^7^ CD3+ T cells were injected i.v. into naive mice.

### Flow cytometry

To confirm depletion of CD4+ and CD8+ cells, whole blood samples (100 μl) were incubated with Fc Block (mouse, BD Biosciences) followed by incubation with fluorochrome-conjugated antibodies (Supplementary Table 3) for 30 minutes in the dark at room temperature (RT). Samples were incubated with RBC Lysis buffer (Thermo Fisher Scientific) before acquisition.

To analyse immune cell infiltration, tumors were harvested and dissociated into single cell suspensions using Tumor Dissociation Kit (mouse, Miltenyi Biotec). Cells were stained with Fixable Viability Dye eFluor506 (Thermo Fisher Scientific), and Fc Block followed by incubation with fluorochrome-conjugated antibodies against surface markers (Supplementary Table 3). Cytokine production was analyzed upon stimulation with Leukocyte Activation Cocktail with GolgiPlug (BD Biosciences). Samples were stained for intracellular markers and cytokines using Foxp3/Transcription Factor Staining Buffer Set (Thermo Fisher Scientific).

Human cells were stained with Zombi NIR Fixable Viability kit (Biolegend), incubated with PBS containing 0.5% bovine serum albumin (Sigma-Aldrich) and 10% FBS for 10 minutes at 4°C, washed and stained with fluorochrome-conjugated antibodies against surface markers (Supplementary Table 3) for 20 minutes at 4°C. Annexin V staining was performed using Annexin V Apoptosis Detection Kit eFluor 450 (Thermo Fisher Scientific) using the manufacturer’s protocol for Annexin V staining and fixable viability dyes. Cytokine production was analyzed using Cyto-Fast Fix/Perm Buffer kit (Biolegend).

Flow cytometry data were acquired using a LSRFortessa X-20 (BD Biosciences) and analyzed using Kaluza (Beckman Coulter) (mouse samples), or BD FACSCanto II (BD Biosciences) or CytoFLEX LX flow cytometer (Beckman Coulter) and analyzed using FlowJo (BD Biosciences, version 10.7.1) (human samples).

### Gene RT-qPCR

Total RNA was isolated from snap frozen tissue and cells using Tissue Lyzer and TRIzol (Thermo Fisher Scientific). 100 ng total RNA was transcribed into single stranded cDNA (Promega) and 1 μl cDNA was used in a 20 μl PCR amplification reaction using SYBR Green master mix (BioRAD). Triplicate reactions were performed for each condition. Target gene expression values were calculated using 2−ΔCt method. ΔCt was calculated by subtracting the geometric mean of the Ct of two reference genes from the Ct of the target genes. Reference genes used were *Ppia* and *Hsp90ab1* for mouse, *GUSB* and *UBC* for human samples, *Ppih* and *Sdha* for the analysis of target engagement in tumors, and *B2m* and *Ppia* for the analysis of target engagement in liver samples. A list of primers is provided in Supplementary Table 4.

### Transfection

For analysis of calreticulin, CD73, and DC maturation, HCT116 were seeded at 150,000 cells/well in 6 well plates (Corning). After 4 hours, cells were transfected by adding 0.5ml of a mix containing 7.5 μl lipofectamine RNAiMAX reagent (Thermo Fisher Scientific) and different concentrations of 1B3 or 3A1 diluted in OPTI-MEM (Thermo Fisher Scientific) to each well. Mock transfected conditions were included for each experiment. Transfected cells were cultured for 1, 3 or 4 days. For analysis of immune cell killing, HCT116 were seeded at 600,000 cells/well in 1.5ml medium and cultured for 8 hours followed by 20–24 hours transfection.

### Isolation of CD14+ cells and DC maturation

CD14+ cells were isolated from PBMC using CD14+ microbeads (human, Miltenyi Biotech). CD14+ cells were counted, a small aliquot was taken to determine purity of CD14+ fraction, and cells were cultured at 1 × 10^6^ cells in 3 ml 10% RPMI in presence of recombinant human (rh)IL-4 (250 IU/ml) and rhGM-CSF (800 IU/ml) (premium grade, Miltenyi Biotech) for 2 days. After 2 days, 1.5 ml culture supernatant was aspirated and replaced with fresh medium containing rhIL-4 (500 IU/ml) and rhGM-CSF (1600 IU/ml). On day 6 of culture, cells were harvested, counted, and a small aliquot was taken to determine DC maturation status. DC were added at 200,000 cells/well in 1ml to HCT116 cells that were mock transfected or transfected with 1B3 or 3A1 for 72 hours. As a positive control for DC maturation, cells were cultured with a cytokine cocktail containing rhIL-1β (200 IU/ml), rhIL-6 (1000 IU/ml), rh-tumor necrosis factor alpha (TNFα) (1000 IU/ml) (premium grade, Miltenyi Biotech), prostaglandin E2 (PGE2) (1 μg/ml) (Stemcell Technologies) (cytokine mix). Cells were cultured for 24 hours and analyzed for DC maturation markers by flow cytometry.

### Co-culture DC and PBMC

PBMC were thawed, counted, and labelled with 2 μM CellTrace Violet (CTV, Thermo Fisher Scientific). After CTV labelling, cells were counted again, resuspended at 2 × 10^6^ cells/ml, and added at DC:PBMC ratio of 1:10 to the co-culture of matured DC and mock, 1B3, or 3A1-transfected HCT116, or DC cultured in presence or absence of cytokine mix. In addition, CTV-labelled PBMC were cultured alone or in presence of Dynabeads Human T-Activator CD3/CD28 for T cell Expansion and Activation (Thermo Fisher Scientific) (ratio beads: PBMC = 1:4 and 1:2). Cells were cultured for 5 days, and Brefeldin A (10 μg/ml, Sigma-Aldrich) was added for the last 18 hours.

### Immune cell killing assay

PBMC-mediated immune cell killing was visualized and quantified using the IncuCyte S3 Live-Cell Analysis System (Sartorius/Essen Bioscience). HCT116 cells were transduced with Incucyte Nuclight Red Lentivirus Reagent (Sartorius) and stable cell lines expressing nuclear restricted mKate2 (red fluorescent protein) were generated using puromycin selection. Cells were mock or 1B3-transfected, harvested, counted, and reseeded into 96 well flat-bottom plates (Corning) at 3,000 cells/well in 100 μl medium. After overnight culture, PBMC were added at tumor cell:PBMC ratio of 1:0, 1:8, and 1:16. For the 1:8 condition, PBMC were added with or without ImmunoCult Human CD3/CD28 T Cell Activator (1 μl per 50,000 cells, Stemcell Technologies). All conditions were analyzed in triplicate or quadruplicate. Co-cultures were monitored and images (9 per well) were captured every 1.5–2 hours at 20× magnification with phase and red channel on.

Analysis was performed using the IncuCyte software (version 2021C). The following analysis definitions for the Basic Analyzer were applied for the phase channel: minimum phase area = 190 μm^2^; and for the red channel: segmentation = surface fit, minimum red area = 40 μm^2^, RCU threshold = 2.0, edge split = on, edge sensitivity = 25. Percentage tumor cell survival was calculated using the average red object counts per mm^2^ of replicate wells for each condition and normalized to tumor cell:PBMC ratio of 1:0 for mock or 1B3-transfected tumor cells.

### Statistical analysis

Statistical analysis was performed using GraphPad Prism V9.2.0. Unpaired, two-tailed *T*-Test, and One- or Two-Way ANOVA with Tukey’s multiple comparisons test were performed as appropriate. The log-rank (Mantel-Cox) test was used to analyse significance of the survival curves. Where used, lines or bar graphs represent mean or median, and error bars represent SD or interquartile range as indicated in figure legends. Box-whisker graphs indicate median and min-max. *P*-values below 0.05 were considered significant and are shown in graphs as ^*^ *p* < 0.05, ^**^ *p* < 0.01, ^***^ *p* < 0.001, ^****^ *p* < 0.0001.

## SUPPLEMENTARY MATERIALS



## Abbreviations

ADP: adenosine diphosphate
AMP: adenosine monophosphate
ATP: adenosine triphosphate
*B2m*: beta-2-microglobulin
CDK4/6: cyclin dependent kinase 4
CRT: calreticulin
CTV: CellTrace Violet
*Cxcl5/9/10*: CXC motif chemokine ligand 5/9/10
DAMPs: damage-associated molecular patterns
DC: dendritic cells
*Dcaf7*: DDB1 and CUL4 associated factor 7
*Entpd1*: ectonucleoside triphosphate dphosphohydrolase 1
*Ezh2*: enhancer of zeste 2 polycomb repressive complex 2 Subunit
FBS: fetal bovine serum
*GUSB*: glucuronidase beta
*Gzmb*: granzyme B
Hsp90ab1: heat shock protein 90 alpha family class B member 1
iDC: immature DC
IFNγ: interferon gamma
LNP: lipid nanoparticle
miRNAs: microRNAs
(m)MDSC: (monocytic) myeloid-derived suppressor cells
moDC: monocyte-derived DC
mRNA: messenger RNA
NK: natural killer
*Nt5e*/*NT5E*: 5′-nucleotidase ecto
PBMC: peripheral blood mononuclear cells
PBS: phosphate buffered saline
PD1: programmed death 1
PGE2: prostaglandin E2
PI3K: phosphoinositide 3-kinase
*Ppia*: peptidylprolyl isomerase A
*Ppih*: peptidylprolyl isomerase H
PTEN: phosphatase and tensin homolog
(rh)IL-4: (recombinant human) interleukin-4
rhGM-CSF: (recombinant human) granulocyte-macrophage colony stimulating factor
RT: room temperature
*Sdha*: succinate dehydrogenase complex flavoprotein subunit A
TME: tumor microenvironment
TNFα: tumor necrosis factor alpha
Treg: regulatory T cells
*UBC*: ubiquitin C
UTRs: untranslated regions
