# Supplementary material for: INT-1B3, an LNP formulated miR-193a-3p mimic, promotes anti-tumor immunity by enhancing T cell mediated immune responses via modulation of the tumor microenvironment and induction of immunogenic cell death
Source: Oncotarget. 2024 Jul 12;15:470–85. doi: 10.18632/oncotarget.28608 (PMC11247534; doi:10.18632/oncotarget.28608)
Supplement: Supplementary file 1 [file oncotarget-15-28608-s001.pdf]

INT-1B3, an LNP formulated miR-193a-3p mimic, promotes anti-tumor immunity by enhancing T cell mediated immune responses via modulation of the tumor microenvironment and induction of immunogenic cell death

SUPPLEMENTARY MATERIALS

Supplementary Methods

Cell lines

The human tumor cell lines HeLa (cervical carcinoma), Hep3B (HCC), SNU449 (HCC), A549 (NSCLC), H460 (NSCLC), H1299 (NSCLC), H1975 (NSCLC), A2058 (melanoma) (ATCC), and Huh7 (HCC, Japanese Cancer Research Resources Bank (JCRB)) were cultured as recommended by the suppliers. For cell passaging, cells were harvested using TripLE Express (Thermo Fisher Scientific).

Stem-loop RT-qPR

INT-1B3 levels in tumor and liver were quantified by determining the level of miR-193a-3p/1B3 (2). Briefly, 100 ng of total RNA was used for the reverse transcription reaction using in-house designed stem-loop (SL) RT primers (IDT). Subsequently, qPCR was performed using SYBR Green (Bio-Rad) and Ct values were interpolated using a calibration curve to determine the amount of 1B3 per ng tissue. It should be noted that the primers used cannot distinguish between endogenous miR-193a-3p expression and the level of exogenous 1B3 due to the guide (antisense) strand sequence being identical. Therefore, it should be assumed that the 1B3 level includes endogenous miR-193a-3p expression, which is determined in PBS control-treated animals. A list of primers is provided in Supplementary Table 3.

3′UTR assay

500 bp gblock gene fragments containing 3′UTR sequences of *Nt5e* and *NT5E* were cloned into psiCHECK-2 plasmids (Promega). 40 ng of each 3′UTR sequence and 2 µg of psiCHECK-2 plasmid was incubated in 1× Cutsmart Buffer (NEB) with 0.5 µl Not1 and 1 µl Pmel at 37°C for 1 hour. Digestion was confirmed by running the product on a 1.5% agarose gel at 100V. The linearized plasmid (6262bp) was excised from the gel, column purified using Nucleospin Gel and PCR Cleanup Kit (Machery-Nagel), and DNA concentration measured using NanoDrop One. To ligate fragments, 12 µl restricted 3′UTR was incubated with 20 ng linear psiCHECK-2 plasmid, 1 µl T4 DNA ligase and 1× Ligase Buffer (Promega) in nuclease free water at 16°C overnight. 25 µl competent DH5α cells were transformed by adding 1–10 ng 3′UTR+psiCHECK-2 ligation mixture. Cells were incubated on ice for 30 minutes followed by 30 seconds heat shock at 42ºC and 2 minutes incubation on ice. Cells were added to LB medium and recovered for 1 hour at 37ºC by shaking at 225 RPM. Cells were then plated on Ampicillin-containing agar plates and incubated overnight at 37ºC. Five colonies from each construct were selected for further analysis by PCR and run on a 1.5% agarose gel to confirm incorporation of 3′UTR into plasmid. Colonies containing the correct inserts were selected for miniprep using NucleoSpin Plasmid Kit (Macherey-Nagel) and sent for sequencing by BaseClear.

HeLa cells were seeded at 50,000 cells/well in 400 µl media in a 24 well plate (Greiner) and cultured overnight. After 24 hours, cells were transfected with 100 ng 3′UTR plasmid using 12 µl Lipofectamine 2000 (Thermo Fisher Scientific). For each 3′UTR plasmid, cells were mock transfected or transfected with 10 nM 3A1 or 1B3. 24 hours after transfection, media was removed, and cells were washed in PBS. 100 µl lysis buffer (Promega) was added to each well and the plate was gently rocked for 15 minutes at RT. 20 µl lysate was transferred to triplicate wells of an opaque 96-well plate. Samples were analyzed using the Dual-Luciferase® Reporter Assay System (Promega) and measured using the Glomax Multi Detection system (Promega). A ratio of Renilla luciferase signal/firefly luciferase signal was calculated and then normalized to mock transfected cells.

Transfection

For analysis of *NT5E* expression, cells were seeded at 400,000 (Hep3B), 500,000 (Huh7), 600,000 (SNU449, H460, H1975), 650,000 (H1299), 750,000 (A549) cells/well in 1.5 ml medium in 6 well plates (Greiner). For analysis of CD73 protein expression by western blot, A2058 cells were seeded at 500,000 cells/well in 6 well plates. After 4 or 24 (Huh7 only) hours of culture, cells were transfected by adding 0.5 ml of a mix containing 7.5 µl lipofectamine RNAiMAX reagent (Thermo Fisher Scientific) and different concentrations of 1B3 or 3A1 diluted in OPTI-MEM (Thermo Fisher Scientific) to each well. Mock transfected conditions were included for each experiment.

For analysis of free phosphate and adenosine levels, A2058 were seeded in flat-bottom 96 well plates (Greiner)

in 100 µl media at 2,000 cells/well or 4,000 cells/well, respectively. After 4 (adenosine) or 24 (free phosphate) hours, cells were mock transfected or transfected with 10 nM 3A1, 1B3, siNT5E or siPool using 0.3 µl Lipofectamine RNAiMAX (Thermo Fisher Scientific). Duplicate wells were included for each condition.

Western blot

Protein extraction and western blot analysis was performed as described [1, 2]. Briefly, approximately 1 × 10^6 cells from mock, 3A1 or 1B3-transfected cells were harvested after 24 hours of transfection and lysed in RIPA lysis buffer (50 mM Tris-HCl pH 8, 150 mM NaCl, 1% NP40, 0.5% sodium deoxycholate, 0.1% SDS, 0.5 mM EDTA) supplemented with protease and phosphatase inhibitor cocktails, and lysates were blotted onto a polyvinylidene difluoride (PVDF) membrane. Membranes were blocked using 5% milk or 5% bovine serum albumin in Tris-buffered saline with Tween (20 mM Tris pH 7.6, 137 mM NaCl, 0.1% Tween), probed overnight with primary antibodies and bound antibodies were visualized using horseradish peroxidase-conjugated (HRP) secondary antibodies (Supplementary Table 4). Proteins were detected using enhanced chemiluminescence (ECL) reagents. αTubulin was used as loading control.

Free phosphate levels

After 24 hours transfection, cells were washed 3× with cell extraction buffer (1 nM CaCl2, 150 mM NaClm 5 mM KCl, 50 nM Tris HCl pH 8.0) before incubation with 500 µM AMP (Sigma-Aldrich) for 30 minutes. For each transfection condition, buffer without AMP was included as control for background phosphate levels. Free phosphate levels were determined using the Malachite Green Phosphate Assay (Sigma-Aldrich) after 24 hours. For each condition, the background was subtracted from the test measurement. Corrected values were normalized to the mock value for each replicate.

Adenosine levels

24 hours after transfection, all media was removed and replaced with fresh warmed media containing adenosine deaminase inhibitor EHNA (15 µM, Sigma-Aldrich) followed by addition of AMP (500 µM) after 1 hour. After 24 hours culture in presence of EHNA and AMP, adenosine levels were measured using the Adenosine Assay Kit (BioVision).

REFERENCES

1. van den Bosch MTJ, Yahyanejad S, Alemdehy MF, Telford BJ, de Gunst T, den Boer HC, Vos RM, Stegink M, van Pinxteren LAH, Schaapveld RQJ, Janicot M. Transcriptome-wide analysis reveals insight into tumor suppressor functions of 1B3, a novel synthetic miR-193a-3p mimic. Mol Ther Nucleic Acids. 2021; 23:1161–71. https://doi.org/10.1016/j.omtn.2021.01.020. [PubMed]
2. Telford BJ, Yahyanejad S, de Gunst T, den Boer HC, Vos RM, Stegink M, van den Bosch MTJ, Alemdehy MF, van Pinxteren LAH, Schaapveld RQJ, Janicot M. Multi-modal effects of 1B3, a novel synthetic miR-193a-3p mimic, support strong potential for therapeutic intervention in oncology. Oncotarget. 2021; 12:422–39. https://doi.org/10.18632/oncotarget.27894. [PubMed]

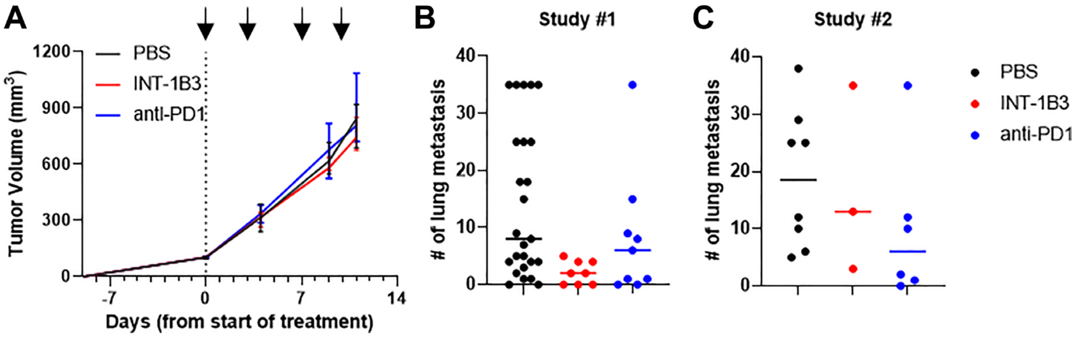

Supplementary Figure 1: Primary tumor volumes in 4T1 model after treatment and number of lung metastasis. (**A**–**C**) Balb/c mice were inoculated orthotopically in the mammary fat pad with murine 4T1 cells (3×10^5). Treatment was initiated when tumors reached a volume of ~100 mm^3 (established tumor, dashed line). Mice were treated BIW with PBS, INT-1B3 (10 mg/kg, i.v.) or anti-PD1 (10 mg/kg, i.p.) for up to seven weeks. Primary tumors were surgically removed when the average tumor volumes per treatment group reached ~800 mm^3. (A) Summary graph showing 4T1 primary tumor volume (mm^3) upon treatment (treatment indicated by black arrows) over time up until primary tumor removal. (B, C) Summary graphs showing number of lung metastasis in the different treatment groups in study #1 (B) (n = 12 per group) and study #2 (C) (n = 30 for PBS and INT-1B3 groups, n = 15 for anti-PD1 group). The number of animals varies between treatment groups because the lung metastases were only counted in mice who did not survive and had to be euthanized due to humane endpoint and not in surviving mice. Lines and error bars indicate median and interquartile range.

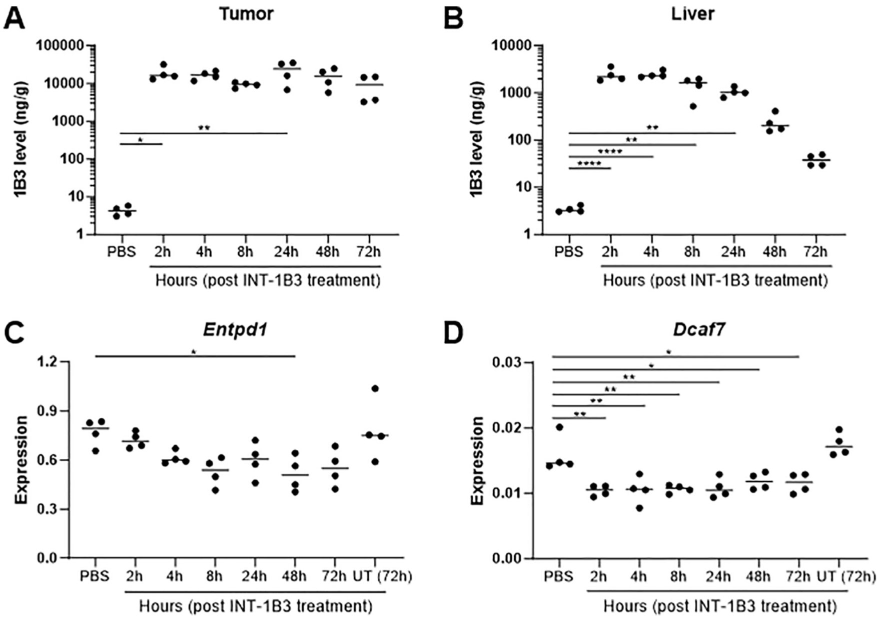

Supplementary Figure 2: 1B3 levels and target expression in tumor and liver. Immunocompetent Balb/c mice bearing 4T1 tumors were randomized and treated with PBS or 10 mg/kg/administration of INT-1B3 (i.v.) once per day for 2 days. Tumors and livers were collected at timepoints indicated after the 2nd administration (n = 4 mice per timepoint). For the PBS-treated group, samples were only collected at 2 hours after the 2nd administration. (**A**, **B**) Summary graphs showing 1B3 levels in tumor (A) and liver (B). 1B3 levels were quantified using Stemloop-RT-qPCR. It should be noted that the primers used cannot distinguish between endogenous miR-193a-3p expression and the level of exogenous 1B3 due to the guide (antisense) strand sequence being identical. Therefore, it should be assumed that the 1B3 level includes endogenous miR-193a-3p expression, which is determined in PBS control-treated animals. (**C**, **D**) Summary graphs showing mRNA expression of INT-1B3 target genes *Entpd1* in tumor (C) and *Dcaf7* in liver (D). For target expression, tumor and liver were also collected from tumor-bearing mice that were untreated for 72 hours post randomization (UT (72 h)). Lines indicate median. Statistical significance was analyzed compared to PBS control. $^*p < 0.05$, $^{**}p < 0.01$, $^{****}p < 0.0001$.

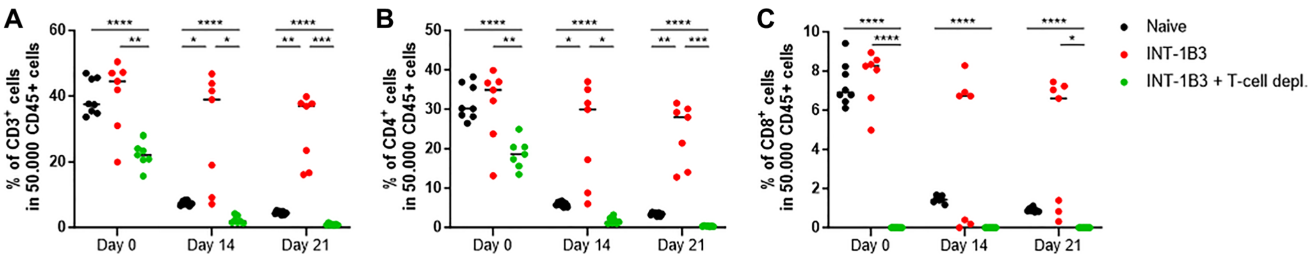

Supplementary Figure 3: Depletion of CD3+, CD4+ and CD8+ T cells upon administration of anti-CD4 and anti-CD8 antibodies. (**A**–**C**) 4T1-challenged, INT-1B3-survivor mice were depleted for T cells as described in Figure 2A. Depletion of T cells in blood of the mice was analyzed by flow cytometry on day 0, 14 and 21 after 4T1 tumor cell rechallenge. Summary graphs showing percentage CD3+ (A), CD4+ (B) and CD8+ (C) T cells within CD45+ cells in each treatment group (n = 7–8 per group). Lines indicate median. Statistical significance was analyzed compared to naive and INT-1B3 treated mice. $^{*}p < 0.05$, $^{**}p < 0.01$, $^{***}p < 0.001$, $^{****}p < 0.0001$.

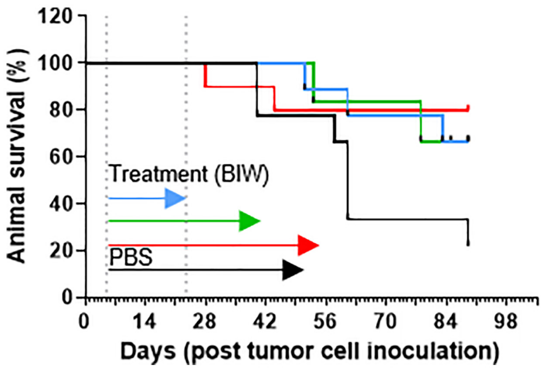

Supplementary Figure 4: Effect of different INT-1B3 administration schedules on animal survival. Experimental design was similar to Figure 1A, except mice were treated with PBS (black line) or INT-1B3 (10 mg/kg, i.v.) until primary tumor resection (blue line), or two weeks (green line) or four weeks (red line) after primary tumor resection. Primary tumors were surgically removed when the average tumor volume per group reached ~800 mm^3. The first dashed line indicates start of treatment and the second dashed line indicates removal of primary tumors. Kaplan-Meier graph showing percentage survival of mice per group at indicated time points (n = 10 per group).

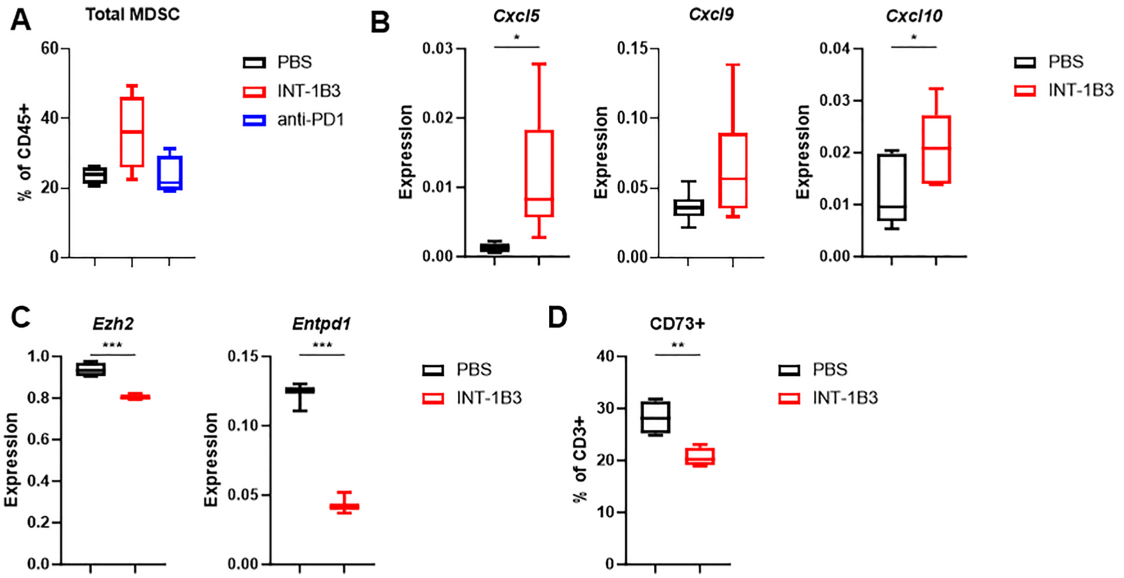

Supplementary Figure 5: Enhanced expression of T cell recruiting chemokines and reduced expression of predicted target genes of miR-193a-3p upon INT-1B3 treatment. Balb/c mice were inoculated orthotopically in the mammary fat pad with murine 4T1 tumor cells (3 × 10^5). Treatment was initiated when tumors reached a volume of ~100 mm^3 (established tumor). PBS, INT-1B3 (10 mg/kg, i.v.) and anti-PD1 (10 mg/kg, i.p.) were administered BIW for up to two weeks (n = 4 per group). Mice were euthanized two days after last injection upon one (week 1) or two (week 2) weeks of treatment, and tumors were harvested for analysis. (**A**) Summary graph showing frequency of total MDSC in tumors from indicated treatment groups (n = 4 per group). Total MDSC were defined as CD45+CD11b+F4/80-. (**B**) Summary graphs showing mRNA expression of *Cxcl5*, *Cxcl9*, *Cxcl10* in tumors from indicated group after two weeks of treatment (n = 6 per group). (**C**) Summary graphs showing mRNA expression of *Ezh2* and *Entpd1* (CD39) in tumors after one week of indicated treatments (n = 6 per group). (**D**) Summary graph showing percentage CD73+ of CD3+ cells in each treatment group. Significance was analyzed compared to PBS, $^{*}p < 0.05$, $^{**}p < 0.01$, $^{***}p < 0.001$. In all graphs, the whiskers indicate min-max, and the lines indicate median.

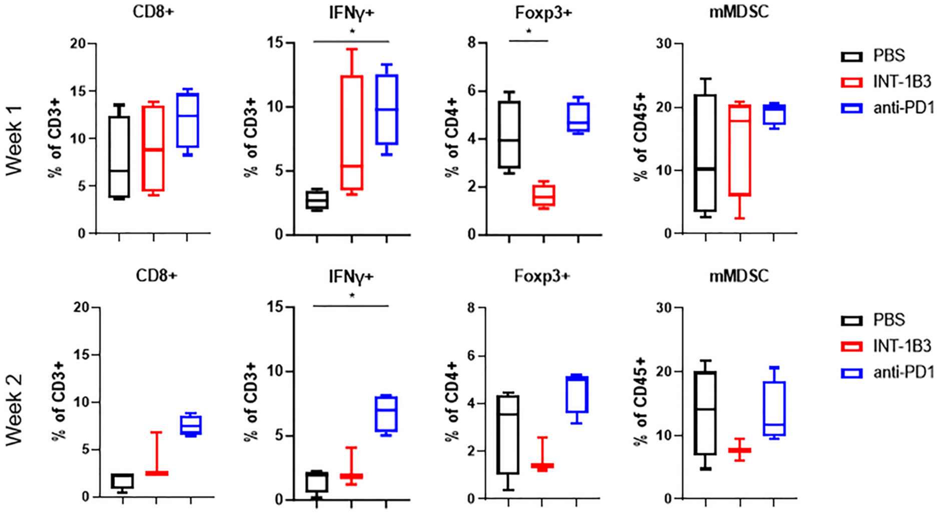

Supplementary Figure 6: Immune cell infiltration in H22 tumors upon INT-1B3 treatment. Balb/c mice were inoculated subcutaneously with murine H22 tumor cells (1 × 10^6). Treatment was initiated when tumors reached a volume of ~100 mm^3 (established tumor). PBS, INT-1B3 (10 mg/kg, i.v.) and anti-PD1 (10 mg/kg, i.p.) were administered BIW for up to two weeks (n = 4 per group). Mice were euthanized two days after last injection upon one (week 1) or two (week 2) weeks of treatment, and tumors were harvested for analysis. Summary graphs showing percentage CD8+ of CD3+, IFNγ+ of CD3+, Foxp3+ of CD4+, and mMDSC in tumors from indicated groups. T cell populations were gated on CD45+CD3+ cells before further analysis of CD4+ and CD8+ populations, and mMDSC were defined as percentage CD11b+Gr-1^{hi+dim} of CD45+. Significance was analyzed compared to PBS, $^{*}p < 0.05$. In all graphs, the whiskers indicate min-max and the lines indicate the median.

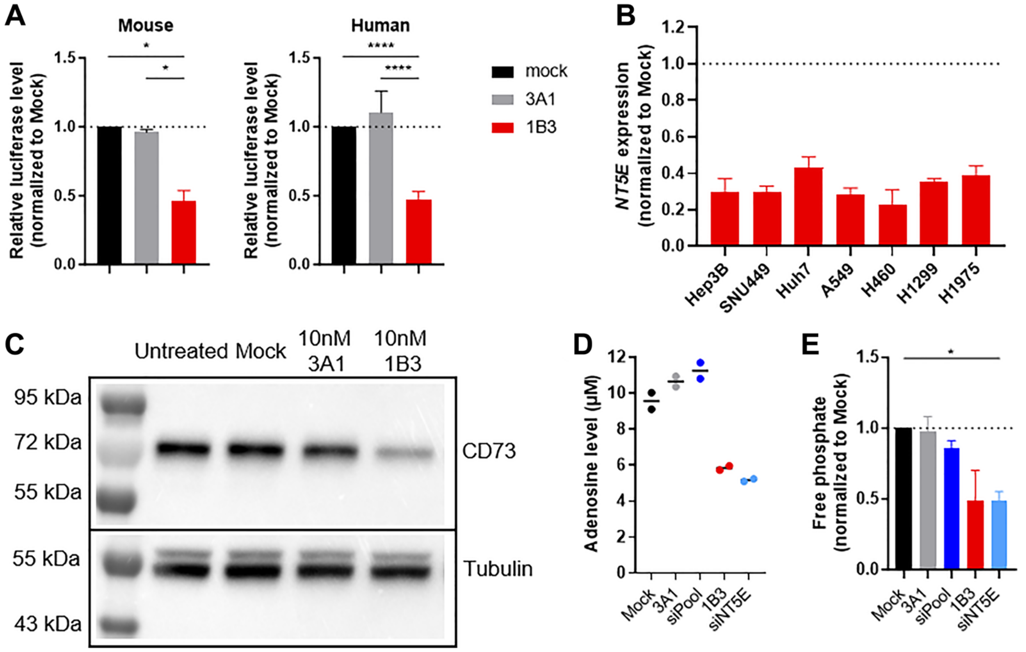

Supplementary Figure 7: 1B3 binds to 3'UTR of NT5E, downregulates CD73 at mRNA and protein level and reduces adenosine production. (**A**) Summary graphs showing relative luciferase levels as a readout for binding of 3A1 and 1B3 to mouse (n = 3) and human (n = 10) *NT5E* normalized to mock. (**B**) Summary graph showing *NT5E* expression in human cancer cell lines upon 24 hours of 1B3 transfection normalized to mock (n = 3). (**C**) Representative western blot showing CD73 protein expression upon 24 hours transfection of A2058 with mock, 3A1 and 1B3. (**D**) Adenosine level after 24 hours transfection of A2058 cells with mock, 3A1, siPool, 1B3 or siNT5E (n = 2). (**E**) Free phosphate level upon 24 hours transfection of A2058 with mock, 3A1, siPool, 1B3 or siNT5E and 30 minutes after administration of AMP normalized to mock (n = 3). Bars or lines indicate mean ± SD, significance was analyzed compared to mock, $^{*}p < 0.05$, $^{****}p < 0.0001$.

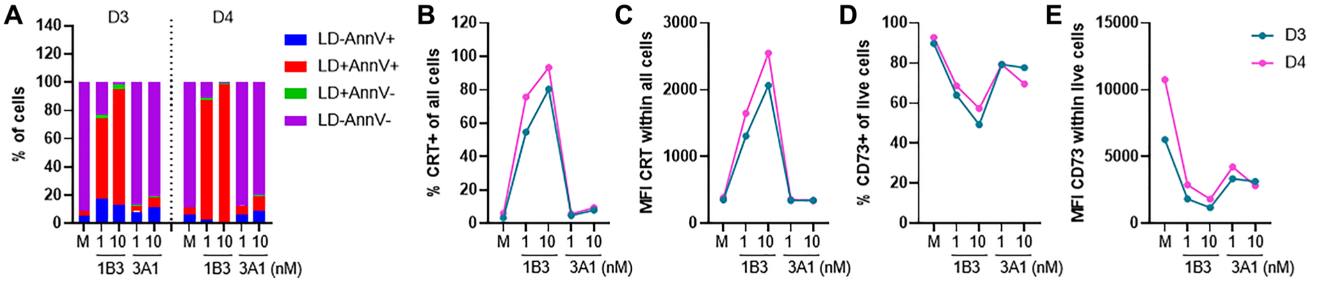

Supplementary Figure 8: Transfection of tumor cells with 3A1 does not induce cell death/apoptosis or upregulation of calreticulin. HCT116 cells were transfected with 1B3 and 3A1 at indicated concentrations or mock (M) transfected and analysed by flow cytometry at various timepoints (n = 1). Mock and 1B3 data is also shown in Figure 5. (**A**) Summary graph showing % LD-AnnV+ (early apoptotic cells), LD+AnnV+ (late apoptotic cells/dead cells), LD+AnnV- and LD-AnnV- (viable cells) cells after 3 and 4 days of transfection with 1B3 or 3A1. (**B**, **C**) Summary graphs showing percentage CRT+ cells (B) and MFI of CRT (C). (D, E) Summary graphs showing percentage CD73+ cells (**D**) and MFI of CD73 (**E**). Summary graphs show mean ± SD.

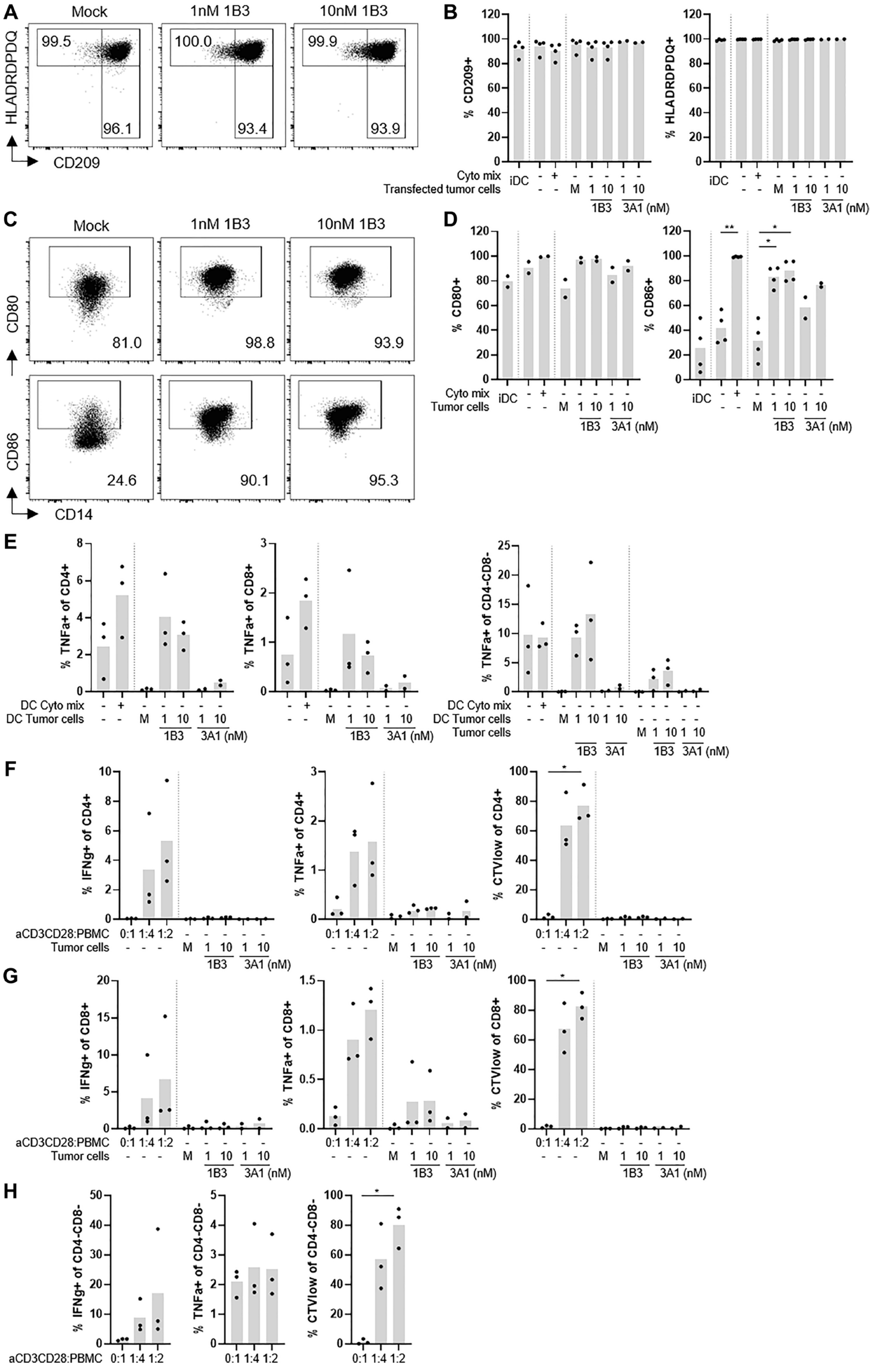

Supplementary Figure 9: 1B3 transfected HCT116 cells do not induce activation of CD4+ and CD8+ T cells. (A–D) HCT116 cells were transfected with 1B3 or 3A1 at indicated concentrations or mock (M) transfected and cultured for 72 hours. CD14+ cells were isolated from PBMC of healthy donors and cultured in presence of rhIL-4 and rhGM-CSF to induce differentiation into immature DC

(iDC) for 6 days. After 6 days, immature DC were harvested and added to transfected tumor cells for 24 hours to induce DC maturation. Immature DC were also cultured in presence or absence of a cytokine cocktail (cyto mix) known to induce DC maturation as a control. Cells were analysed for expression of DC (maturation) markers by flow cytometry before and after maturation ($n = 2–4$). (A, B) Representative dot plots (A) and summary graphs (B) showing percentage CD209+ and HLA-DR, DP, DQ+ cells. (C, D) Representative dots plots (C) and summary graphs (D) showing percentage CD80+ and CD86+ cells gated on CD45+ cells. (**E**–**G**) CTV- labelled PBMC were co-cultured for 5 days with DC matured by co-culture with HCT116 transfected with mock, 1B3 or 3A1 at a ratio of DC:PBMC of 1:10, or with transfected HCT116 cells ($n = 2–3$). (E) Summary graphs showing percentage TNFα+ of CD4+, CD8+, and CD4-CD8- T cells. $n = 2–3$. (F, G) CTV-labelled PBMC were stimulated with αCD3CD28 coated beads at a ratio of beads:PBMC of 1:0, 1:4 and 1:2, or co-cultured with HCT116 transfected with mock, 1B3 or 3A1. Summary graphs showing percentage IFNγ+, TNFα+ and CTVlow of CD4+ cells (F) and CD8+ T cells (G). (**H**) Summary graphs showing percentage IFNγ+, TNFα+ and CTVlow of CD4-CD8- T cells upon stimulation with αCD3CD28 coated beads. $^{*}p < 0.05$, $^{**}p < 0.01$. Bars in summary graphs represent mean.

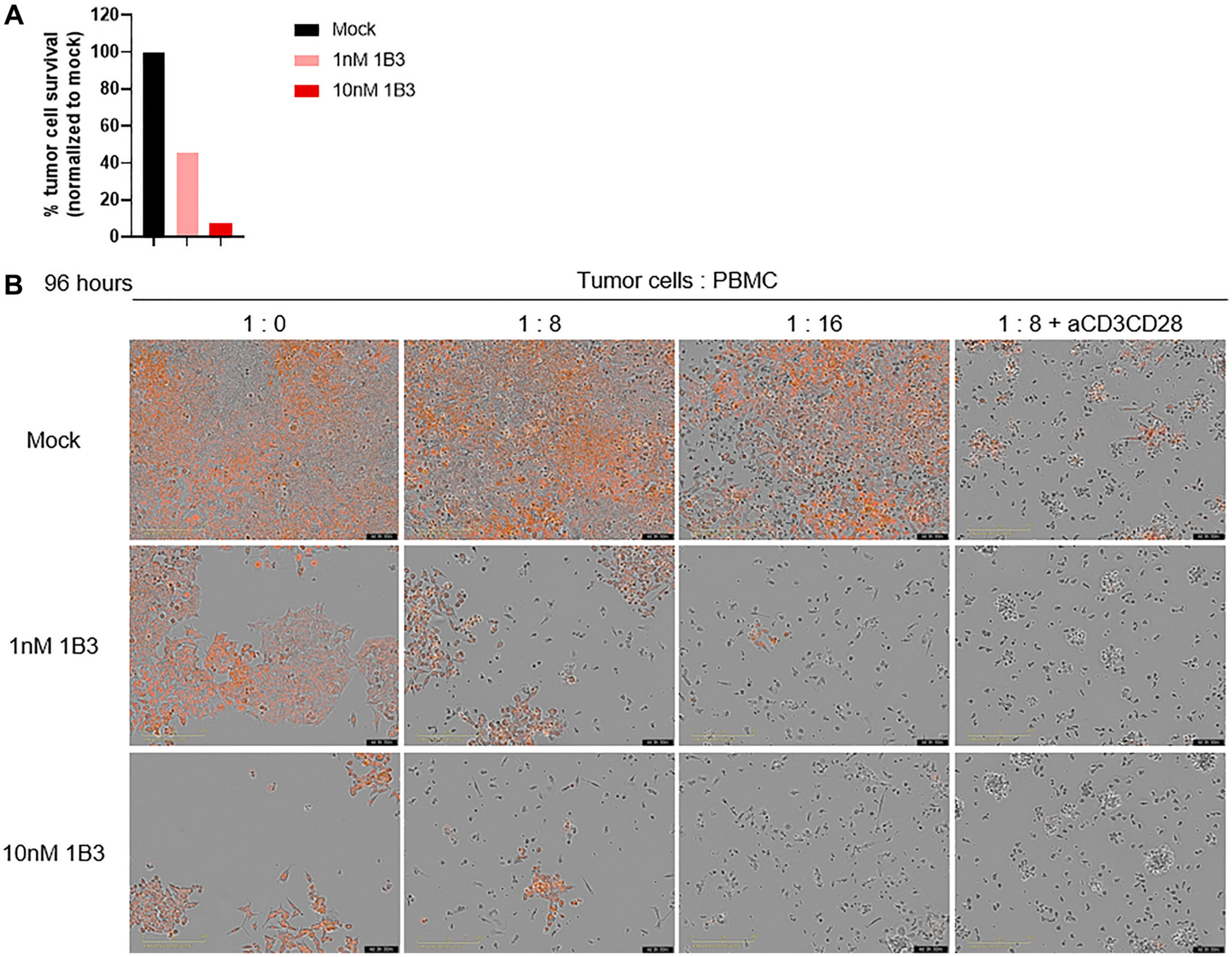

Supplementary Figure 10: PBMC-mediated cytotoxicity against 1B3 transfected tumor cells. NucLight Red labelled HCT116 cells were transfected overnight with 1B3 at indicated concentrations or mock transfected. After overnight transfection, cells were harvested, counted, reseeded into 96-well plates and allowed to adhere to the plate overnight-24 hours before adding PBMC. PBMC were added at ratio tumor cells:PBMC of 1:0, 1:8, 1:16 or 1:8 + αCD3CD28. Data shown is one representative of 3 independent experiments. (**A**) Summary graph showing percentage tumor cell survival upon 1B3 transfection. (**B**) Representative images showing PBMC mediated immune cell killing of tumor cells at 96 hours after adding PBMC.

Supplementary Table 1: Western blot antibodies

Marker	Clone	Company	Catalog no.
NT5E/CD73	D7F9A	Cell Signaling Technologies	13160
αTubulin	4G1	Santa Cruz	sc-58666
Goat-anti-Rabbit IgG-HRP	N/A	Cell Signaling Technologies	7074
Goat-anti-Mouse-IgG-HRP	N/A	Santa Cruz	sc-2005

Supplementary Table 2: Oligonucleotide sequences with limited 2’-O-methyl nucleotide modification on the passenger strand

Oligonucleotide	Sequence 5′–3′
1B3 passenger (sense) strand	UGGGACUUUGUAGGCCAGUUTT
1B3 guide (anti-sense) strand	AACUGGCCUACAAAGUCCCAGU
3A1 passenger (sense) strand	UAACGACGCGACGACGUAATT
3A1 guide (anti-sense) strand	UUACGUCGUCGCGUCGUUATT

Supplementary Table 3: Flow cytometry antibodies

Anti-mouse antibodies				
**Marker**	**Fluorochrome**	**Clone**	**Company**	**Catalog no.**
CD45	PerCPCy5.5	30-F11	Biolegend	103132
CD3	FITC	17A2	BD Biosciences	561798
CD4	APC	RM4-4	Biolegend	116014
CD8	PE	53–6.7	Biolegend	100708
CD45	BUV661	30-F11	BD Biosciences	565079
CD3	BUV395	17A2	BD Biosciences	740268
CD4	APC-eFluor780	GK1.5	Thermo Fisher Scientific	47-0041-82
CD8	eFluor610	53–6.7	Thermo Fisher Scientific	61-0081-82
FOXP3	PE	FJK-16S	Thermo Fisher Scientific	12-5773-82
F4/80	PE/CY7	BM8	Biolegend.	123114
CD11b	FITC	M1/70	Biolegend.	101206
Gr-1	AF700	RB6-8C5	Biolegend.	108422
LAG3 (CD223)	BV650	C9B7W	BD Biosciences	740560
IL-2	BV421	JES6-5H4	Biolegend	503825
IFNγ	PerCPCy5.5	XMG1.2	Biolegend	505822
CD73	APC	TY/11.8	Biolegend	127210
Live/Dead	eFluor506	N/A	Thermo Fisher Scientific	65-0866-14
Anti-human antibodies				
**Marker**	**Fluorochrome**	**Clone**	**Company**	**Catalog no.**
IgG1	DyLight 488	MOPC-21	Enzo Life Sciences	ADI-SAB-600-488-F
Calreticulin	DyLight 488	FMC 75	Enzo Life Sciences	ADI-SPA-601-488-F
CD73	APC	REA804	Miltenyi Biotech	130-111-909
Annexin V	eFluor 450	N/A	Thermo Fisher Scientific	48-8006-69
Live/Dead	Zombi NIR	N/A	Biolegend	423106
CD3	BV510	UCHT1	Biolegend	300448

CD3	AF700	UCHT1	BD Biosciences	557943
CD14	Alexa Fluor 488	63D3	Biolegend	367130
CD86	BV421	BU63	Biolegend	374212
CD209	PE	REA617	Miltenyi Biotech	130-117-706
CD83	PE-Vio770	REA714	Miltenyi Biotech	130-110-505
CD80	PE	2D10	Biolegend	305208
HLA-DR,DP,DQ	APC	Tü39	Biolegend	361714
CD45	Alexa Fluor 700	2D1	Biolegend	368514
CD45	PerCP	2D1	Biolegend	368506
CD4	AF488	RPA-T4	Biolegend	300519
CD8a	APC	HIT8a	Biolegend	300912
CD45RO	PE-Cy7	UCHL1	Biolegend	304230
IFNγ	PE	4S.B3	Biolegend	502509
TNFα	BV605	MAb11	Biolegend	502936

Supplementary Table 4: Primers

Gene	Mouse/human		Primer sequence 5′–3′
B2m	Mouse	Forward	CTGACCGGCCTGTATGCTATC
		Reverse	AGGCGGGTGGAACTGTGTTA
Cxcl5	Mouse	Forward	CCAGAAGGAGGTCTGTCTGGAT
		Reverse	CACTGGCCGTTCTTTCCACT
Cxcl9	Mouse	Forward	CGAGGCACGATCCACTACAA
		Reverse	AGTCCGGATCTAGGCAGGTT
Cxcl10	Mouse	Forward	TCTGAGTGGGACTCAAGGGAT
		Reverse	ATTCTCACTGGCCCGTCATC
Dcaf7	Mouse	Forward	GACTAGAGACCGGGCAAGTG
		Reverse	CGGCTGAAGGCGATGTCATA
Entpd1	Mouse	Forward	GCCGAATGCATGGAACTGTC
		Reverse	CTGCCGATTGTTCGCTTTCC
Ezh2	Mouse	Forward	GTCGCCTCGGTGCCTATAA
		Reverse	GATCCAGAACTTCATCCCCCA
Gzmb	Mouse	Forward	CAATCAGATATGTGCGGGGGA
		Reverse	TTTTACACACAAGCGGGCCT
GUSB	Human	Forward	TGCGTAGGGACAAGAACCAC
		Reverse	GGGAGGGGTCCAAGGATTTG
Hsp90ab1	Mouse	Forward	AGATTCCACTAACCGACGCC
		Reverse	CCGCACTCGCTCCACAAA
NT5E	Human	Forward	AGGCCTTTGAGCATAGCGTG
		Reverse	CTCTGTCTCCAGGTTTTCGGG
Ppia	Mouse	Forward	GCGGCAGGTCCATCTACG
		Reverse	GCCATCCAGCCATTCAGTC
Ppih	Mouse	Forward	AATCGAGCTCTTTGCAGACG
		Reverse	TATCCTATCGGAACGCCATC

Sdha	Mouse	Forward	GAGGAAGCACACCCTCTCAT
		Reverse	GGAGCGGATAGCAGGAGGTA
UBC	Human	Forward	CAGCCGGGATTTGGGTCG
		Reverse	CACGAAGATCTGCATTGTCAAGT
miR-193a-3p	Mouse	SL-RT primer	GTCGTATCCAGTGCAGGGTCCGAGGT ATTCGCACTGGATACGACACTGGGA
miR-193a-3p	Mouse	Forward	TGCCCGAACTGGCCTACAAAGT
		Reverse	GTGCAGGGTCCGAGGT
